# VCP at the crossroads of UPS and autophagy in cancer

**DOI:** 10.1016/j.gendis.2026.102264

**Published:** 2026-05-28

**Authors:** Charlie J. Hitchman, Julia I. Phillips, Hee-Won Park, Hua Lu

**Affiliations:** a Department of Biochemistry & Molecular Biology and Tulane Cancer Center, Tulane University School of Medicine, New Orleans, LA 70112, USA; b School of Medicine, Tulane University, New Orleans, LA 70112, USA

**Keywords:** Autophagy, Cancer, p53, Ubiquitin-proteasome system, VCP/p97

## Abstract

Proteostasis is maintained by the coordinated action of the ubiquitin–proteasome system (UPS) and autophagy–lysosome pathways. Valosin-containing protein (VCP/p97), an AAA + unfoldase, sits at its intersection by extracting ubiquitinated clients and assembling cofactor-defined complexes that determine substrate fate. In cancer, VCP up-regulation and altered cofactor recruitment rewire these ubiquitin-dependent routing decisions. By recruiting E3 ligases and deubiquitinases that remodel K48- and K63-linked ubiquitin chains, VCP biases substrates toward Ufd1–Npl4-coupled proteasomal turnover or autophagy-linked clearance, enabling oncogene stabilization or tumor suppressor loss. These principles are reflected in cancer axes that modulate autophagy flux, invasion and metastasis, PI3K/AKT/mTOR signaling, and immune evasion. The tumor suppressor p53 illustrates this complexity with state-dependent outcomes: VCP promotes proteasomal turnover of wild-type p53 through the canonical Ufd1–Npl4 complex, whereas it can stabilize the R273H hotspot mutant in a chaperone/holdase-like manner, prolonging gain-of-function phenotypes. Opposing regulators further control VCP function: PLAC8 enhances VCP–Ufd1–Npl4 activity and is linked to wild-type p53 turnover, PI3K/AKT/mTOR activation, and context-dependent autophagy effects. In contrast, SVIP can outcompete other VCP cofactors and, via acylation-dependent membrane targeting, redirect VCP toward lysosome-associated functions with predominantly tumor-suppressive effects. Collectively, this framework motivates therapeutic strategies that modulate VCP–cofactor interactions to regulate substrate degradative fate. Approaches include disrupting the Ufd1–Npl4 axis, biasing VCP from UPS to autophagy, altering subcellular localization, and reprogramming VCP for targeted proteolysis, with implications for cancer, fibrosis, neurodegeneration, and multisystem proteinopathy.

## Introduction

Proteostasis is maintained primarily by the ubiquitin–proteasome system (UPS)^1^^,^^2^ and autophagy–lysosome pathways,^3^^,^^4^ which collaborate to remove short-lived or misfolded proteins and larger structures such as aggregates.^5^^,^^6^ UPS is the process by which proteins destined for degradation are recognized, modified through the addition of polyubiquitin chains, and then hydrolyzed into peptides or individual amino acids at the 26S proteasome.^7^ Ubiquitin modifications can adopt many forms.^8^ The two most common types are K48- and K63-linkages, where K48/K63 refers to the ubiquitin lysine residue to which the next ubiquitin in the chain is added. K48-linked ubiquitins are a key marker for proteins destined for proteasomal degradation,^9^ whereas K63-linked ubiquitins signal for autophagic degradation of the marked protein.^10^^,^^11^ Autophagy collectively refers to three different branches of autophagic pathways: macroautophagy, microautophagy, and chaperone-mediated autophagy.^12^ Macroautophagy delivers cytosolic proteins to the lysosome first through the formation of a double membrane-bound vesicle, called the autophagosome. The autophagosome fuses with the lysosome to form an autolysosome, and proteins are subsequently degraded by lysosomal proteases.^3^^,^^13^ This review discusses the role of valosin-containing protein (VCP) in UPS and macroautophagy, referred to subsequently as autophagy, in cancer with direct relevance to neurodegenerative and fibrotic diseases.

**Figure 1:**
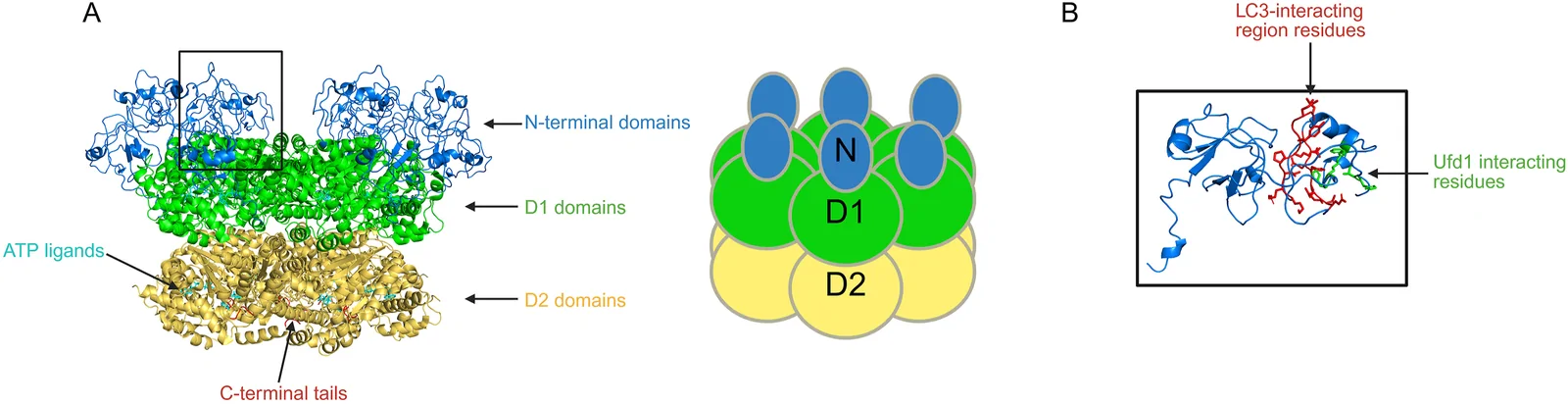
The domain structure of the VCP hexamer and interacting regions of the VCP N-terminal domain. **(A)** The hexameric VCP structure (PBD ID: 5FTN^126^) in the ATP-bound (N-terminal domain “up”) conformation is displayed in cartoon representation with ATP analogue molecules displayed as sticks (cyan). VCP N-terminal domains (residues 1–186) are colored blue, D1 domains (residues 208–459) are colored green, D2 domains (residues 481–761) are colored yellow, and C-terminal tails (residues 761–806, only 761–768 displayed) are colored red. The black box highlights a single VCP N-terminal domain. **(B)** Structure of a single VCP N-terminal domain in cartoon representation, colored blue. LC3-interacting region (LIR) residues are colored red with side chains displayed as sticks: LIR 1 sequence – ^139^LEAYRPIR^146^; LIR 2 sequence – ^159^AVEFKVVE^166^.^40^ Ufd1 interacting residues are colored green with side chains displayed as sticks: R113, H115, F131, I182, and H183.^148^ Models are displayed using PyMOL.^149^ All cartoons are created in https://BioRender.com.

VCP, also commonly known as p97, is a member of the extended family of ATPases associated with various cellular activities (AAA+) family^14^ and utilizes ATP hydrolysis to unfold and extract proteins from larger complexes and membranes.^15^^,^^16^ VCP functions in all compartments of the cell and has been implicated in UPS and autophagy,^17^, ^18^, ^19^ but also in other lesser-known branches of “phagy” in maintaining organelle dynamics, function, and turnover.^20^ VCP, a 806 amino acid protein with a molecular weight of 90 kDa, contains three domains: the N-terminal domain, D1 domain, and D2 domain, as well as linker regions between the domains and a short C-terminal tail^21^ (Fig. 1A). The N-terminal domain can adopt multiple conformations and is predominantly involved in protein–protein interactions with substrates or VCP cofactors. VCP assembles into a homohexamer with six-fold pseudo-symmetry, enabled by the intermolecular interactions between D1 and D2 domains.^22^ D1 and D2 are the ATPase domains, both containing Walker A and Walker B motifs responsible for binding and hydrolyzing ATP.^23^ ATP hydrolysis initiates substantial conformational changes in the D2 domain, which provide the force to segregate or unfold client proteins.^23^^,^^24^ The mechanism of client unfolding and processing is discussed and reviewed elsewhere.^25^^,^^26^

VCP functions are mostly established through interactions with ubiquitinated proteins, either directly or indirectly mediated by cofactors (Fig. 1B). VCP involvement in UPS relies on the formation of a ternary complex of VCP and two cofactors, ubiquitin recognition factor in ER-associated degradation 1 (Ufd1) and nuclear protein localization 4 (Npl4),^27^ binding to VCP N-terminal domain via their short hydrophobic peptide (SHP)-binding motif and ubiquitin regulatory X-like (UBXL) domain, respectively. These are two motifs/domains present in VCP–cofactor interactions, with other relevant interactions including VCP-interacting motifs (VIMs) and VCP-binding motifs (VBMs) (extensively reviewed by Hänzelmann and Schindelin 28). Simultaneous binding of multiple cofactors, as well as competition between cofactors binding to the N-terminal domain via the same types of interactions, regulates the functions of VCP.^29^ The VCP–Npl4–Ufd1 ternary complex recognizes the polyubiquitinated substrate, before unfolding it and passing it directly onto the proteasome for degradation.^30^ Crucially, the Ufd1–Npl4 heterodimer displays a strong preference for polyubiquitinated rather than monoubiquitinated substrates, as well as stronger affinity for K48 ubiquitin linkages compared with other linkage types.^31^^,^^32^ The mechanism of VCP–Ufd1–Npl4 ternary complex formation, as well as substrate recognition and selectivity, has been discussed and reviewed extensively.^31^^,^^33^^,^^34^

VCP plays multifaceted roles in autophagy of individual proteins and protein aggregates, acting both at initiation and during autophagosome/lysosomal fusion.^35^^,^^36^ At early stages, VCP promotes the formation of the phagophore by stabilizing the initiating phosphatidylinositol 3-kinase (PtdIns3K) complex.^37^ Inhibition of VCP causes accumulation of lysosomal-associated membrane protein (LAMP)-like 1/2-positive vacuoles, suggesting a role for VCP in the late stage of lysosomal fusion.^38^ VCP also mediates clearance of aggregate-prone proteins implicated in neurodegenerative diseases, such as mutant Huntingtin (HTT) in Huntington's disease. This process involves interaction of mHTT with the VCP N-terminal domain, as well as interaction of VCP with the autophagic factor microtubule-associated protein 1 light chain 3 (LC3) via its LC3-interacting regions (LIRs).^39^^,^^40^ Gossypol acetate, despite inhibiting VCP ATPase activity, enhances VCP–mHTT–LC3 ternary complex formation, promoting mHTT degradation.^41^ Furthermore, VCP is required for both formation and clearance of aggresomes (cytoplasmic inclusions encased by intermediate filaments) by autophagy.^42^ The C-terminal tail mediates interaction with histone deacetylase 6 (HDAC6),^43^ while the N-terminal domain engages UBX-domain-containing protein 1(UBXN1) and Ufd1–Npl4 (which preferentially bind to K48-linked ubiquitin)^44^^,^^45^ HDAC6, in contrast, binds to K63-linked ubiquitinated proteins (*e*.*g*., Parkinson disease protein 7).^46^

VCP dysfunction is linked to numerous human diseases, with strong associations to neurodegenerative diseases such as multisystem proteinopathy (MSP1), also known as inclusion body myopathy associated with Paget's disease of bone and frontotemporal dementia (IBMPFD).^47^^,^^48^ The cause of VCP-linked neurodegenerative disorders is predominantly genetic, with both loss-of-function^49^^,^^50^ and gain-of-function mutants causing disease phenotypes.^36^^,^^51^^,^^52^ In this review, we will instead focus on how dysregulation of VCP-mediated protein degradation contributes to cancer, although the conclusions drawn have significant implications for neurodegenerative and fibrotic diseases.

## VCP in cancer

Compared with neurodegenerative diseases caused by VCP mutations, VCP dysfunction in cancer most often results from its overexpression in tumor cells relative to normal cells. VCP has been implicated in a wide range of cancers with consistent mechanisms and phenotypic outcomes. These have been established through *in vitro* and *in vivo* experiments involving overexpression, knockdown, or pharmacological inhibition. Constantini et al (2021) provided a comprehensive overview of the oncogenic VCP mechanisms identified at the time, as well as the cancer types in which these mechanisms are found.^53^ Building from this, Table S1 provides an overview of the common cancer-causing mechanisms of and phenotypes associated with VCP upregulation discovered since, as well as highlighting potential therapeutic strategies in targeting VCP. As well as providing a summary, the major aim of this review is to outline a common theme. That is, VCP–cofactor interactions guide the degradative fate of many cancer proteins to either UPS or autophagy, with dysregulation leading to aberrant degradation or stabilization and subsequently cancer. Initially, this will be discussed in the context of altered ubiquitination status caused by interactions with E3 ubiquitin ligases and deubiquitinases, followed by discussion with two lesser-known interactors of VCP: small VCP-interacting protein (SVIP) and placenta-specific gene 8 protein (PLAC8).

## VCP regulates the degradation of cancer-associated proteins through alterations in their ubiquitination status

**Figure 2:**
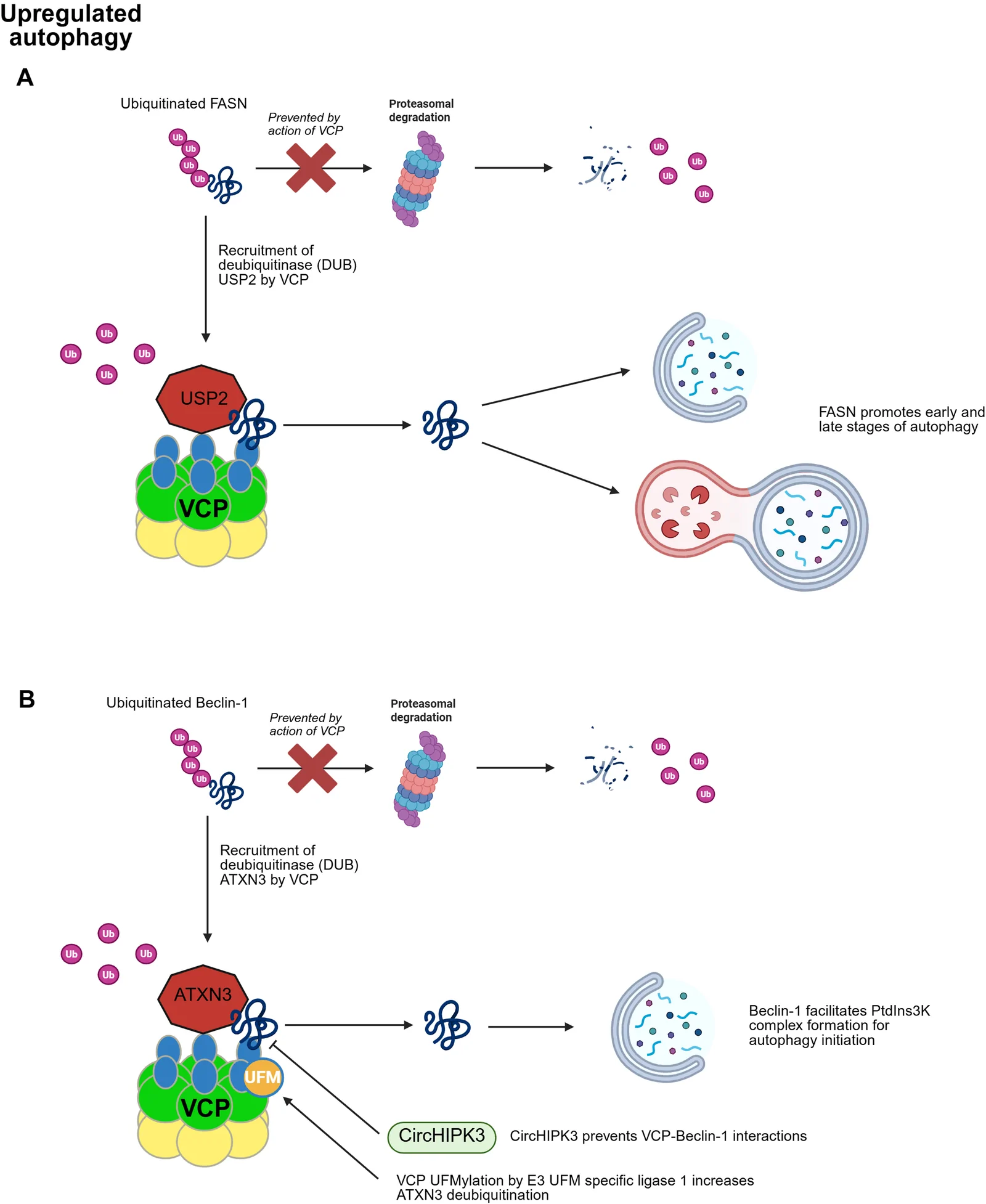
Regulation by VCP of cancer-associated protein ubiquitination and effects on autophagy. The red cross denotes the fate of the protein of interest in the absence of VCP action. **(A)** Ubiquitinated FASN is degraded by the proteasome. VCP recruits deubiquitinase (DUB) USP2 to deubiquitinate FASN. Stabilized FASN promotes early and late stages of autophagy. **(B)** Ubiquitinated Beclin-1 is degraded by the proteasome. VCP recruits ATXN3 to deubiquitinate Beclin-1. Stabilized Beclin-1 promotes autophagy initiation through formation of the PtdIns3K complex. Circular RNA HIPK3 inhibits Beclin-1/VCP interactions. VCP UFMylation enhances the Beclin-1/VCP/ATXN3 axis.

**Figure 3:**
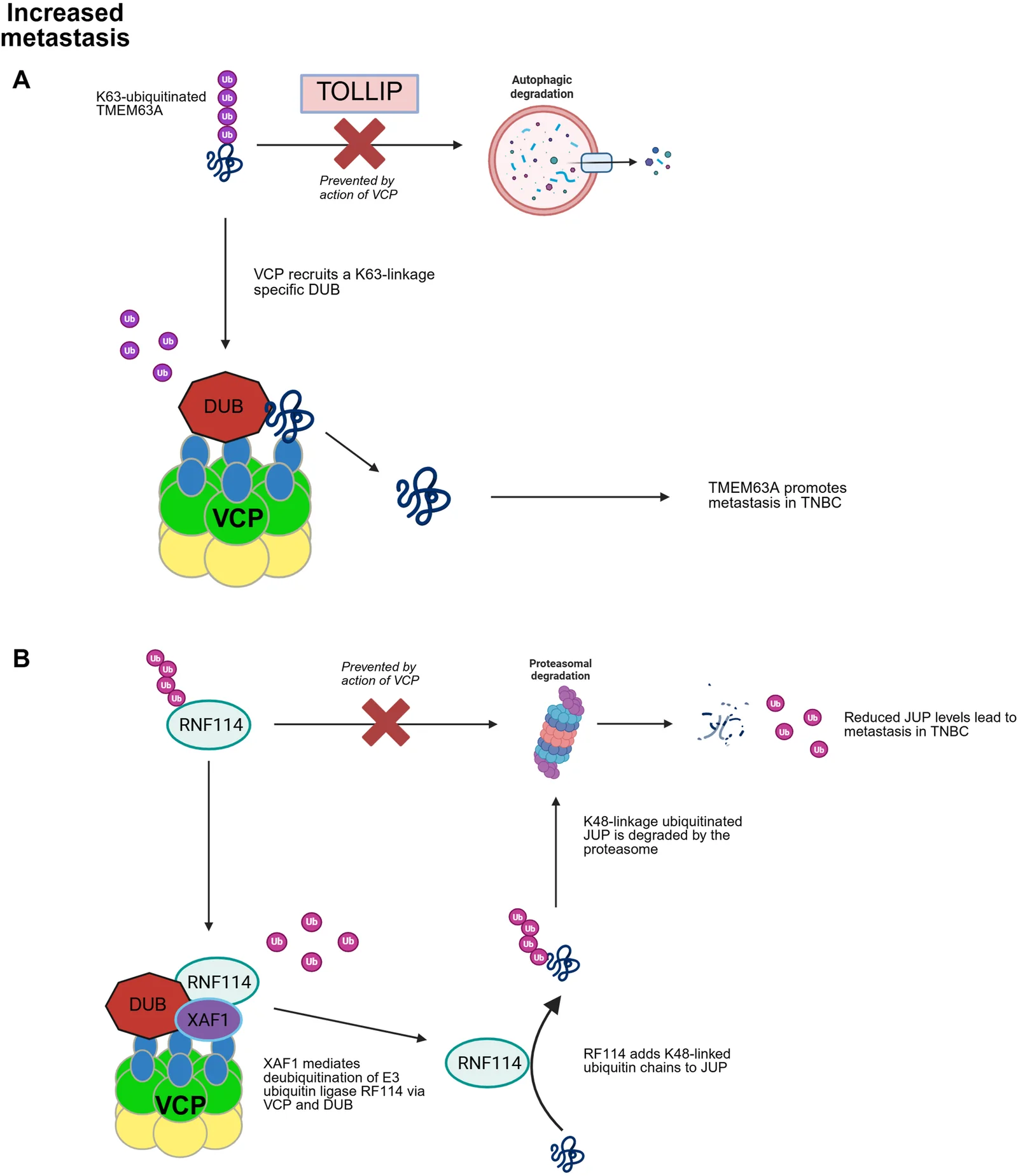
Regulation by VCP of cancer-associated protein ubiquitination and effects on cancer cell metastasis. The red cross denotes the fate of the protein of interest in the absence of VCP action. **(A)** Ubiquitinated RNF114 is degraded by the proteasome. XAF1 promotes deubiquitination of RNF114 via VCP and an unknown DUB. Stabilized RNF114 ubiquitinates JUP with K48-linked ubiquitin, promoting JUP proteasomal degradation and triple-negative breast cancer (TNBC) metastasis. **(B)** K63-linkage ubiquitinated TMEM63A is degraded by autophagy via TOLLIP. VCP recruits a K63-linkage specific DUB to deubiquitinate TMEM63A. Stabilized TMEM63A promotes TNBC metastasis.

**Figure 4:**
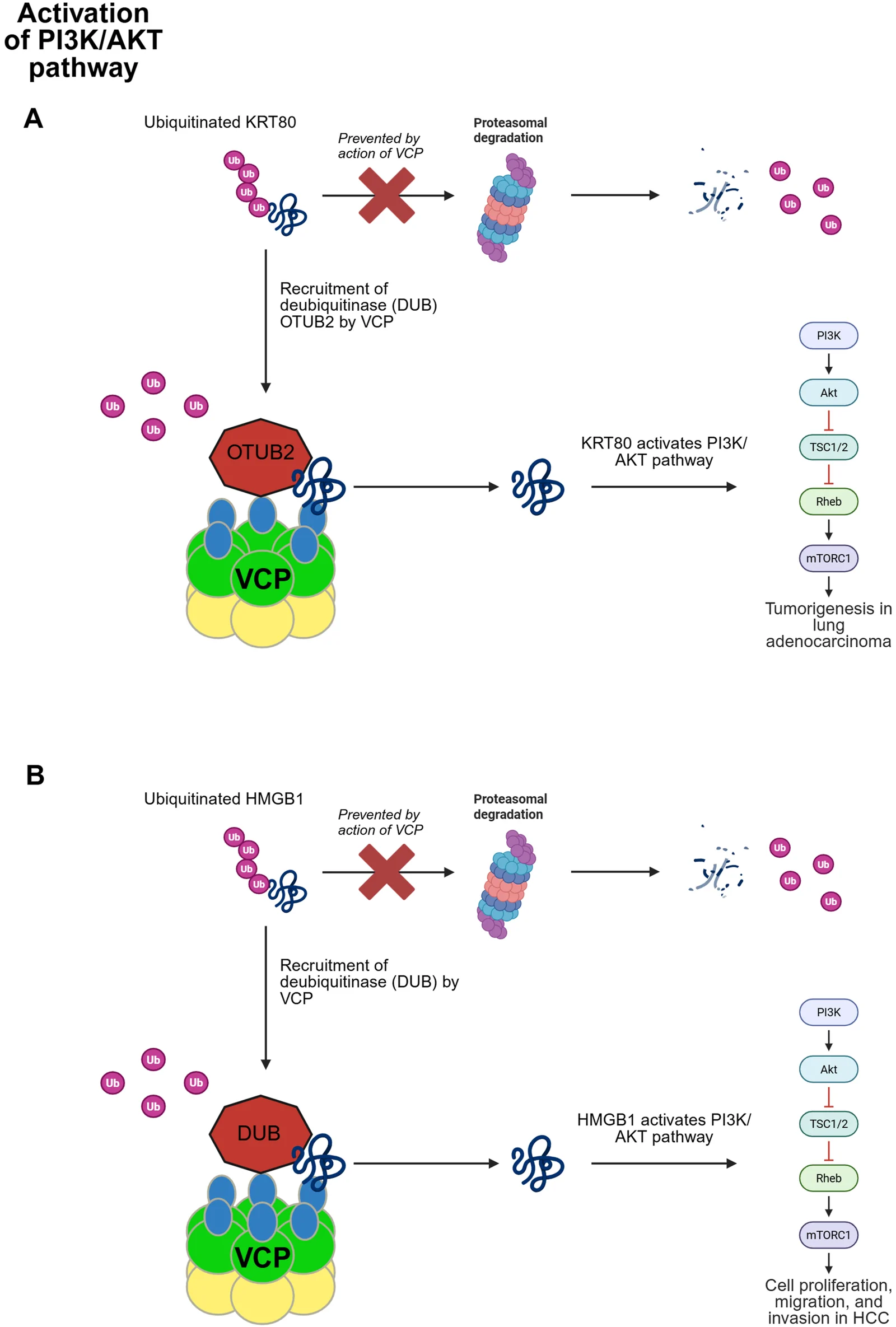
Regulation by VCP of cancer-associated protein ubiquitination and effects on PI3K/AKT pathway activation. The red cross denotes the fate of the protein of interest in the absence of VCP action. **(A)** KRT80 is degraded by the proteasome. VCP recruits OTUB2 to deubiquitinate KRT80. Stabilized KRT80 activates the PI3K/AKT pathway, leading to tumorigenesis in lung adenocarcinoma. **(B)** Ubiquitinated HMGB1 is degraded at the proteasome. VCP likely recruits an unknown DUB to deubiquitinate HMGB1. Stabilized HMGB1 activates the PI3K/AKT pathway, leading to cell proliferation, migration, and invasion in hepatocellular carcinoma (HCC).

**Figure 5:**
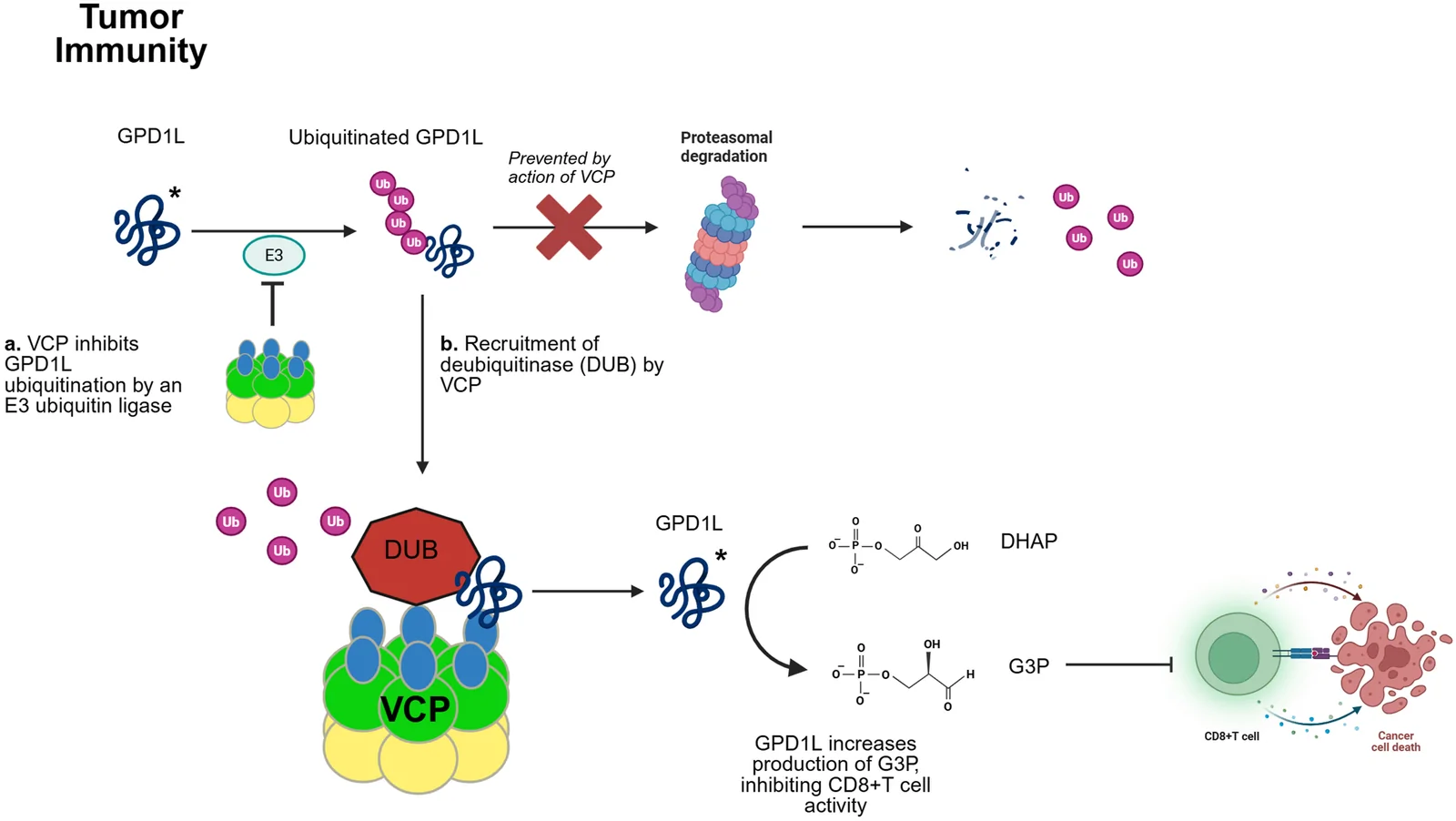
Regulation by VCP of cancer-associated protein ubiquitination and effects on tumor immune evasion. The red cross denotes the fate of the protein of interest in the absence of VCP action. Ubiquitinated GPD1L is degraded by the proteasome. VCP action leads to reduced GPD1L ubiquitination, likely via either a) inhibiting ubiquitination or b) recruiting a DUB. Stabilized GPD1L catalyzes the production of G3P from DHAP, which inhibits the activity of the CD8^+^ T cells in the hepatocellular carcinoma (HCC) tumor microenvironment.

VCP influences the fate of key cancer-related proteins by modulating their ubiquitination status. Although VCP is neither an E3 ubiquitin ligase nor a deubiquitinase (DUB), it can interact with both types of enzymes through its N-terminal domain. These VCP-enzyme interactions are common in many different cancer phenotypes associated with VCP dysregulation, as well as across many cancer subtypes (overview provided in Figure 2, Figure 3, Figure 4, Figure 5 and Table 1). Firstly, in osteosarcoma, co-immunoprecipitation with mass spectrometry identified ubiquitin-specific protease 2 (USP2) and fatty acid synthase (FASN) as VCP-associated proteins in 143B and U2OS cells.^54^ Protein-stability and ubiquitination assays supported that VCP stabilized FASN by promoting USP2-dependent removal of K48-linked ubiquitin and preventing proteasomal degradation. This VCP–USP2–FASN axis increased autophagy flux and supported osteosarcoma growth *in vitro* and in 143B xenograft and orthotopic models (Fig. 2A). A similar autophagy-stimulating effect was seen in bladder cancer via VCP-mediated stabilization of Beclin-1. Initially, protein-stability and ubiquitination assays in HeLa cells indicated that VCP promoted Ataxin-3 (ATXN3)-dependent deubiquitination and stabilization of Beclin-1 (BECN1), supporting PtdIns3K complex function and early autophagy initiation.^55^ In bladder cancer models, co-immunoprecipitation and immunofluorescence showed that circHIPK3 RNA bound to VCP and weakened the VCP–BECN1 interactions, suppressing autophagy in T24 and EJ cells and reducing tumor growth in a T24 xenograft model.^56^ Furthermore, circHIPK3 levels were discovered to be significantly down-regulated in bladder cancer tissues, highlighting the importance of its tumor-suppressive function. In parallel, co-immunoprecipitation with mass spectrometry identified VCP as a substrate of UFM-specific ligase 1, which adds a ubiquitin-fold modifier (UFM) moiety to VCP lysine-109.^57^ This modification leads to increased ATXN3-mediated deubiquitination of BECN1, likely via stabilization of the ternary complex, and supports autophagy initiation. This suggests a potential pro-tumorigenic effect of VCP UFMylation (Fig. 2B).

**Table 1:** Regulation of protein degradative fate by VCP and ubiquitin-modifying enzyme interactions as illustrated in Figure 2.

Cancer phenotype	Ubiquitin-modifying enzyme	Protein with altered fate	Effect of the enzyme	Adaptor protein	Cancer type	Figure 2 panel	Key reference
Upregulated autophagy	USP2	Fatty acid synthase (FASN)	Reduced K48-linked Ub; stabilizing	N/A	Osteosarcoma	A	54
Upregulated autophagy	ATXN3	Beclin-1	Reduced Ub; stabilizing	N/A	Bladder	B	55, 56, 57
Increased metastasis	Unknown DUB	TMEM63A	Reduced K63-linked Ub; prevents autophagic degradation	TOLLIP	Breast	C	58
Increased metastasis	Unknown DUB	RNF114	Reduced Ub; stabilizing	XAF1	Colorectal	D	59
Increased metastasis	RNF114	JUP	Increased K48-linked Ub; promotes proteasomal degradation	N/A	Colorectal	D	59
Activating PI3K/AKT pathway	OTUB2∗	KRT80	Reduced Ub; stabilizing	N/A	Lung	E	60,61
Activating PI3K/AKT pathway	Unknown DUB	HMGB1	Reduced Ub; stabilizing	N/A	Liver (HCC)	F	62
Tumor immunity	Unknown E3 ligase/DUB	GPD1L	Reduced Ub at lysine 228; stabilizing	N/A	Liver (HCC)	G	63

Note: PI3K, phosphoinositide 3-kinase; USP2, ubiquitin-specific protease 2; ATXN3, ataxin-3; DUB, deubiquitinase; RNF114, ring finger protein 114; OTUB2, ovarian tumor deubiquitinase, ubiquitin aldehyde binding 2; TMEM63A, transmembrane protein 63A; JUP, junction plakoglobin; KRT80, keratin 80; HMGB1, high mobility group box protein 1; GPD1L, glycerol-3-phosphate dehydrogenase 1-like protein; Ub, ubiqutination; HCC, hepatocellular carcinoma. The asterisk (∗) indicates that there is evidence for the action of OTUB2 with KRT80 and the action of KRT80 and VCP, but the combined action of VCP and OTUB2 is speculative.

Conversely, VCP stabilizes transmembrane protein 63A (TMEM63A), a tetra-spanning membrane protein, by preventing TMEM63A autophagic degradation, with pro-tumorigenic effects.^58^ Interaction proteomics and co-immunoprecipitation identified TMEM63A in a complex with VCP. Pharmacological inhibition of VCP by CB-5083 or genetic depletion in breast cancer cells (MDA-MB-231, BT547) led to decreased levels of TMEM63A, with levels rescued by the autophagy inhibitor bafilomycin-A1. This indicates that VCP opposes TMEM63A autophagic turnover. Knockdown of autophagy receptor toll-interacting protein (TOLLIP) led to a significant increase in TMEM63A levels. TOLLIP belongs to a class of autophagy proteins that recognize their cargo through interaction with K63-linked ubiquitin molecules. These results suggest that VCP may stabilize TMEM63A by recruiting a K63-linkage-specific DUB, reducing K63-linked ubiquitination, recognition by TOLLIP, and subsequent autophagic degradation (Fig. 3A). This VCP-dependent TMEM63A stabilization mechanism was linked to pro-migratory/invasive triple-negative breast cancer behavior *in vitro* (MDA-MB-231, BT549) and to increased tumor growth and lung metastasis *in vivo* (MDA-MB-231 xenograft and tail-vein metastasis models).^58^ Increased metastasis caused by VCP-mediated protein stabilization was also seen in colorectal cancer. Co-immunoprecipitation and proteomic interaction mapping placed VCP within an XIAP-associated factor 1 (XAF1)–ring finger protein 114 (RNF114) complex, where XAF1 acts as a novel adaptor for VCP, facilitating VCP-mediated deubiquitination of the E3 ligase RNF114.^59^ This E3 ligase then promotes K48-linked ubiquitination and proteasomal degradation of junction plakoglobin (JUP). which leads to increased migration and metastasis (Fig. 3B). This axis was linked to increased migration in colorectal cancer cell models (RKO, DLD-1, SW480, HCT116) and increased lung colonization *in vivo* using luciferase-labeled MC38 cells.

Many of the proteins with fates influenced by VCP are activators of the phosphoinositide 3-kinase (PI3K)/protein kinase B (AKT) pathway, another common VCP cancer feature. In lung adenocarcinoma cells (A549), keratin-80 (KRT80) was identified as a VCP-interacting protein by co-immunoprecipitation with mass spectrometry and immunofluorescence co-localization.^60^ VCP knockdown decreased KRT80 protein stability in cycloheximide-chase experiments, consistent with loss of deubiquitination-dependent protection from proteasomal degradation. KRT80 overexpression partially mitigated the inhibitory effects of VCP knockdown on PI3K/AKT pathway activation,^60^ suggesting that KRT80 is downstream of VCP in lung cancer and contributes to PI3K/AKT-linked malignant phenotypes (Fig. 4A). While the specific DUB coupling KRT80 deubiquitination to VCP has not been identified in lung cancer, ovarian tumor deubiquitinase, ubiquitin aldehyde binding 2 (OTUB2) deubiquitinates and stabilizes KRT80 in gastric cancer,^61^ activating AKT signaling, suggesting a plausible mechanism by which VCP could coordinate DUB–substrate interactions to promote KRT80 stabilization. The same methods were also used to identify high mobility group box 1 protein (HMGB1) as a VCP interactor. Cycloheximide-chase and ubiquitination assays supported that VCP stabilized HMGB1 by reducing ubiquitin–proteasome degradation, likely via recruitment of a DUB.^62^ HMGB1 depletion blunted, and HMGB1 re-expression rescued, VCP-dependent PI3K/AKT/mechanistic target of rapamycin (mTOR) activation and associated malignant phenotypes in hepatocellular carcinoma models (Huh7, MHCC-LM3) (Fig. 4B). Ubiquitination and proteasome-inhibition experiments also support VCP stabilization of glycerol-3-phosphate dehydrogenase 1-like protein (GPD1L) in hepatocellular carcinoma, via the ubiquitin–proteasome pathway, with lysine-226 implicated as a key regulatory ubiquitination site.^63^ Reciprocal co-immunoprecipitation and GST pull-down assays supported direct VCP–GPD1L binding, consistent with a model in which VCP regulates GPD1L K226 ubiquitination by coordinating access of an E3 ligase or a DUB (Fig. 5). VCP-dependent GPD1L stabilization increased extracellular G3P, which suppressed CD8^+^ T-cell function in hepatocellular carcinoma models (HCCLM3, Hepa1-6-OVA/OT-1 co-culture) and was linked to immunosuppressive tumor growth *in vivo*.

## VCP differentially regulates the degradation of wild-type and mutant p53

p53 is a stress-responsive transcription factor that preserves genome integrity by coordinating cell-cycle arrest, senescence, apoptosis, ferroptosis, DNA repair, and metabolic reprogramming, with transcriptional control as its core mode of action.^64^^,^^65^ Transcriptional activity of p53 requires the formation of a homotetramer to interact with p53-specific promoter regions. *TP53* is the most frequently mutated gene in human cancer; most alterations are single-missense “hotspot” mutations clustering in the DNA-binding domain.^66^ These include both DNA-contact mutants and structural mutants, many of which exert dominant-negative and gain-of-function effects that promote tumor progression.^67^, ^68^, ^69^ Dominant negative effects arise when tetramers of p53 consist of both wild-type and mutant p53 and are unable to bind to DNA promoters. In this regard, the activity of wild-type p53 is negated by the presence of mutant p53 within a tetramer. Gain-of-function effects occur due to aberrant interactions enabled by alternate conformations of p53 mutants compared with wild-type p53. In normal cells, wild-type p53 abundance and activity are tightly controlled by ubiquitin-mediated turnover. The E3 ligase mouse double minute 2 homolog (MDM2) targets p53 for proteasomal degradation with support from partner E3 ligase murine double minute X (MDMX).^70^ This process is counteracted by the action of acetylases that modify key p53 lysine residues to prevent MDM2–p53 interaction.^71^ In contrast to wild-type, many p53 mutants are stabilized by chaperones, leading to mutant p53 accumulation.^72^, ^73^, ^74^ Targeting the degradation of mutant p53 has become an attractive approach to reduce the oncogenic dominant negative and gain-of-function effects to treat mutant p53 cancers.^75^, ^76^, ^77^, ^78^

### VCP suppresses wild-type p53 activity

The interaction between VCP and wild-type p53 was first discovered in non-small cell lung carcinoma. VCP is significantly overexpressed in these cancers,^79^ and is associated with suppression of apoptosis, increased cell cycle progression and proliferation, and increased metastasis.^80^, ^81^, ^82^ Initial studies identifying this interaction also showed that VCP overexpression increased levels of tumor-associated proteins, nuclear factor kappa B (NFκB), nuclear response factor 2 (Nrf2), and sirtuin-1 (SIRT1), while decreasing levels of wild-type p53. Elevated NFκB levels and activation of the NFκB pathway were mediated by VCP through enhanced proteasomal degradation of inhibitor of kappa B (IκB).^82^ Coimmunoprecipitation confirmed the mechanistic role of VCP, showing direct interaction with both IκB and wild-type p53. Inhibition of VCP with the small molecule eeyarestatin I reversed these effects. This study provided the first evidence for VCP acting as a direct mediator of wild-type p53 proteasomal degradation. Given that proteasomal degradation mediated by VCP requires the ubiquitin-recognizing cofactors Ufd1 and Npl4,^27^ it was anticipated that this would also be the case for VCP processing of wild-type p53.

**Figure 6:**
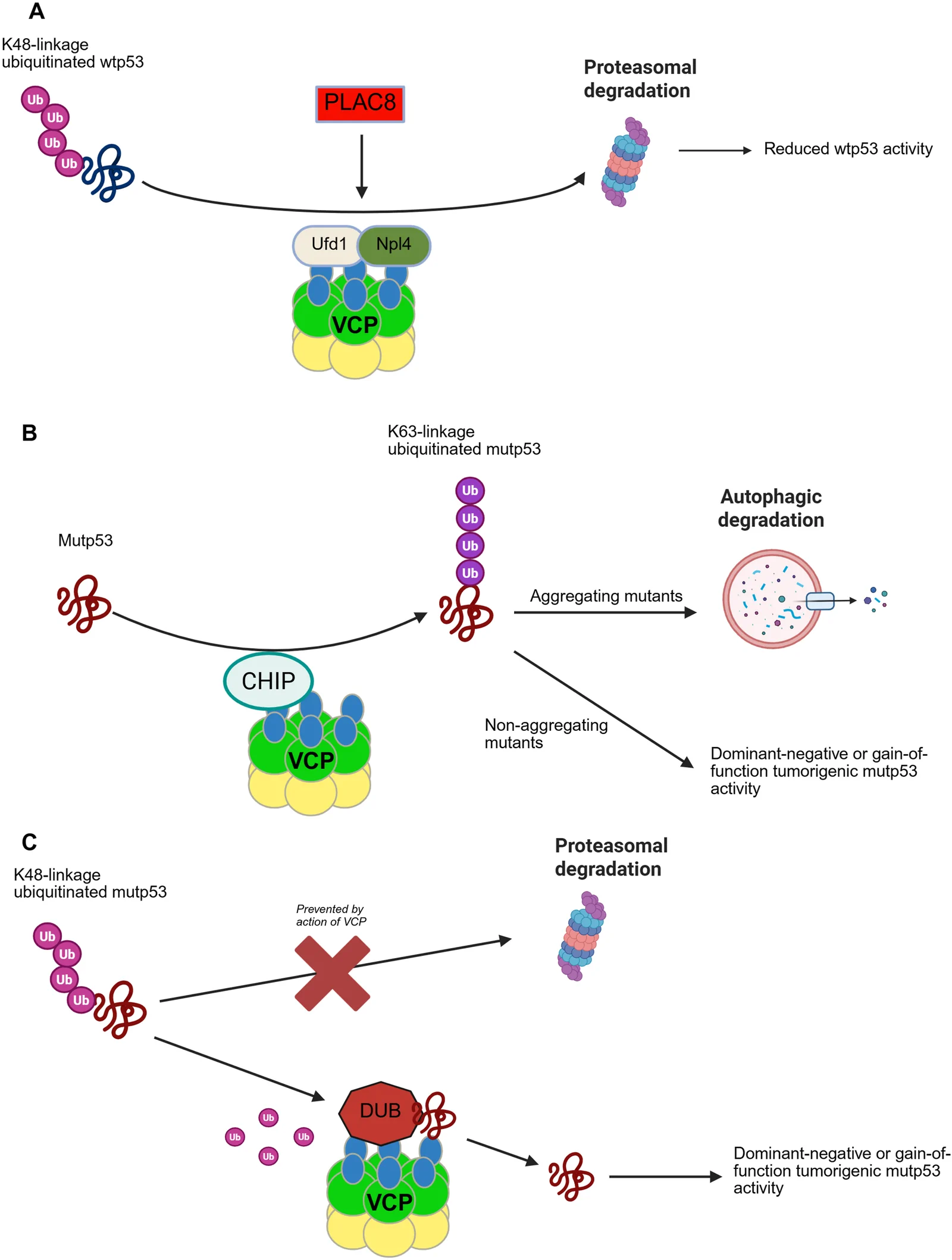
Regulation of wild-type and mutant p53 degradation by VCP. VCP interacts with cofactors via its N-terminal domains, promoting alternate fates of wild-type and mutant p53. **(A)** Ubiquitin chains linked by K48 residues (K48 linkages) on wild-type p53 are recognized by VCP cofactors Ufd1 and Npl4 and processed by VCP before being passed onto the proteasome for degradation. The action of PLAC8 stimulates this process, likely via recruitment of cofactors to VCP, increasing degradation of wild-type p53. **(B, C)** Mutant p53 is protected from proteasomal degradation by VCP, either (B) through the action of an E3 ubiquitin ligase such as CHIP which promotes alternate K63-linked ubiquitination (which can lead to autophagic degradation for aggregating mutants in hypoxic conditions), or (C) through the action of a deubiquitinase (DUB), such as USP7 or ATXN3, which removes K48-linked ubiquitin molecules required for Ufd1/Npl4 recognition. The red cross denotes the putative fate of K48-linked ubiquitinated mutp53 in the absence of VCP action.

A recent study by Sun et al used co-immunoprecipitation and mass spectrometry to identify the interaction of VCP, Ufd1, Npl4, and p53.^83^ Knockdown of VCP and Ufd1 by siRNA inhibited degradation of wild-type p53 in a non-small cell lung carcinoma cell line (A549), leading to accumulation of ubiquitinated p53, confirming the importance of ubiquitination and ubiquitination recognition. Furthermore, they identified an accessory protein that activates this process. PLAC8 expression levels regulated VCP-mediated wild-type p53 degradation, with PLAC8 overexpression and knockdown leading to reduced or increased wild-type p53 levels, respectively (Fig. 6A). In the context of lung fibrosis, increased PLAC8 activity reduced wild-type p53 function and led to decreased apoptosis and cellular senescence. Both of these processes accelerate fibrosis progression, indicating that their downregulation by PLAC8 mitigates fibrosis. In this study, PLAC8 transcription was shown to be reduced in type 2 alveolar epithelial-like cells, leading to fibrosis. Conversely, PLAC8 has previously been identified as an oncogene with multiple tumorigenic effects.^84^ This interaction with VCP, as further discussed below, may provide an explanation for some of PLAC8's tumorigenic effects.

### VCP promotes mutant p53 activity

Most studies identifying the role of VCP in p53 degradation have focused on wild-type p53,^82^^,^^83^^,^^85^ with strong evidence that elevated VCP levels reduce p53 activity. However, the opposite effect has been seen with a hotspot DNA-binding domain mutant in pancreatic cancer by our lab.^86^ Specifically, Wang et al showed that VCP interacted with the p53 R273H mutant, identified in a pancreatic ductal adenocarcinoma cell line (PANC-1). The R273H mutant is classified as a DNA contact mutant, in which the mutation weakens p53–DNA interactions such that it can no longer act as a transcription factor for canonical p53 target genes.^87^ This contributes to the dominant-negative effect on wild-type p53 whilst also leading to specific gain-of-function effects.^67^ Particularly, this mutant has been shown to promote the transcription of oncogenes *CDK1*, *MAP2K3*, and *NFΚB2*. Wang et al identified VCP as a binding partner of this mutant p53 through p53 antibody co-immunoprecipitation and mass spectrometry analyses. This aligns with findings for wild-type p53;^82^^,^^83^ however, upon VCP knockdown, mutant p53 levels decreased rather than increased. This finding suggests that VCP stabilizes mutant p53 and, if absent, mutant p53 is subject to alternate degradation. A similar phenomenon has been seen with mutant p53 and members of the heat shock protein families in which mutant p53 degradation is prevented by chaperone stabilization.^72^^,^^73^ Thus, in this instance, VCP may display a chaperone- or holdase-like activity, likely recognizing alternate mutant conformations of p53 well-established in the literature.^88^, ^89^, ^90^ Mutant p53/VCP interactions were also confirmed by pulldown assays for other hotspot mutants: R175H, Y220C, and R248W, although the cellular effects of these interactions were not investigated. The behavior of VCP as a holdase or chaperone was also observed in another study on prion-like protein stabilization,^91^ and is consistent with the recognition and stabilization of mutant Huntingtin by VCP's N-terminal domain.^40^

Decreases in mutant p53 levels induced by VCP knockdown or knockout were accompanied by G2/M cell cycle arrest, increased apoptosis, and reduced cancer cell survival.^86^ Knockdown of mutant p53 produced the same effect and reduced transcription of gain-of-function targets associated with cell survival, thus showing directly that VCP stabilization of mutant p53 promotes oncogenic effects. MDM2 levels are typically low in mutant p53 cell lines, as wild-type p53 activity is required for MDM2 gene transcription.^92^^,^^93^ This suggests MDM2 is not the endogenous E3 ubiquitin ligase of mutant p53, but, as discussed, VCP is known to interact with other E3 ubiquitin ligases, which may be responsible for the altered ubiquitination status of mutant p53 relative to wild-type p53. One E3 ubiquitin ligase stands out: the C terminus of Hsc70-interacting protein (CHIP), also commonly known as STIP1 homology and U-box containing protein 1 (STUB1). CHIP is well established as an E3 ubiquitin ligase, which ubiquitinates both wild-type and mutant p53.^74^^,^^94^ Furthermore, its U-box domain provides intrinsic chaperone activity and enables it to selectively recognize misfolded or aggregated proteins,^95^ such as p53 mutants. A study by Maan et al further demonstrated that CHIP was able to ubiquitinate wild-type p53 and aggregating mutants of p53 (R110L, R110P, and R175H), promoting their degradation. However, they also showed that the type of ubiquitination linkages varied with p53 variant, and that this affected the degradation pathway. CHIP added K48-linked ubiquitin molecules to wild-type p53, which promoted proteasomal degradation, whereas aggregated mutant p53 was modified by K63-linked ubiquitin molecules and degraded by autophagy. VCP and CHIP are known to interact, with disruption of this interaction leading to reduced proteasomal degradation of VCP substrates.^96^^,^^97^ The effect of CHIP on p53 ubiquitination was exacerbated in hypoxic and starvation conditions in cell lines spanning many cancer types. Thus, CHIP may be the alternate E3 ubiquitin ligase that promotes mutant p53 stabilization mediated by VCP (Fig. 6B). However, this study also found that CHIP expression had no effect on non-aggregating p53 mutant degradation, such as R273H, in these conditions. This suggests that an alternate VCP cofactor may be involved other than CHIP.

As discussed for FASN, Beclin-1, KRT80, and RNF114, VCP mediates altered ubiquitination through recruitment of DUBs, which remove ubiquitin molecules and promote stabilization. Thus, stabilization of mutant p53 mediated by VCP could be due to the action of DUBs instead of or as well as altered activity of E3 ubiquitin ligases (Fig. 6C). Two DUBs stand out as likely candidates based on their interactions with both VCP and p53. The first is USP7 (also known as HAUSP). USP7 directly deubiquitinates wild-type p53, leading to a reduction in proteasomal degradation and increased p53 activity.^98^ More recently, USP7 has also been shown to promote stability of mutant p53 and maintain cell stemness in colorectal cancer.^99^ In addition, the interplay between USP7 and VCP is prominent in the nucleus, where ubiquitination levels regulate DNA replication. In the absence of USP7, DNA replication can become blocked, and VCP, with its cofactor Fas-associated factor 1 (FAF1), is required to extract ubiquitinated proteins.^100^ Furthermore, USP7-mediated deubiquitination of nucleotide excision repair component XPC prevents binding to VCP and its cofactors and reduces proteasomal degradation.^101^ The second DUB candidate is ATXN3. Like USP7, ATXN3 was shown to by Liu et al to act on wild-type p53, increasing stability and activity.^102^ Moreover, this study used mass spectrometry to show that both wild-type p53 and VCP were pulled down with ATXN3 from cell lysates. This is direct evidence for the simultaneous interaction of ATXN3 with wild-type p53, and VCP with ATXN3, which also leads to wild-type p53 deubiquitination. Notably, however, a recent study by Lange et al showed that ATXN3 acted as a debranching enzyme of VCP substrates, removing K63-linked ubiquitin moieties and leaving a homotypic K48-linked chain that is more suitable for VCP processing and proteasomal degradation.^103^ If ATXN3 activity were unable to debranch mutant p53 compared with wild-type, this would explain the difference in their cellular fates, as regulated by the binding of both proteins to VCP.

Further investigation into the effects of VCP-interacting proteins is important to identify how these regulators affect the degradation of p53 and potentially influence the mechanisms by which VCP causes cancer. We will now highlight the interactions between VCP and PLAC8 and between VCP and SVIP, and their effects on proteasomal degradation and autophagy implicated in disease.

## Differential regulation of VCP cancer-associated effects by PLAC8 and SVIP

### VCP and PLAC8

As previously mentioned, PLAC8 has been identified as an activator of VCP with interaction of Ufd1 and Npl4.^83^ Elevated PLAC8 levels lead to increased degradation of wild-type p53 mediated by VCP. Before the interaction with VCP was discovered, PLAC8 was already established as an oncoprotein and possible drug target for treating cancer.^84^

**Figure 7:**
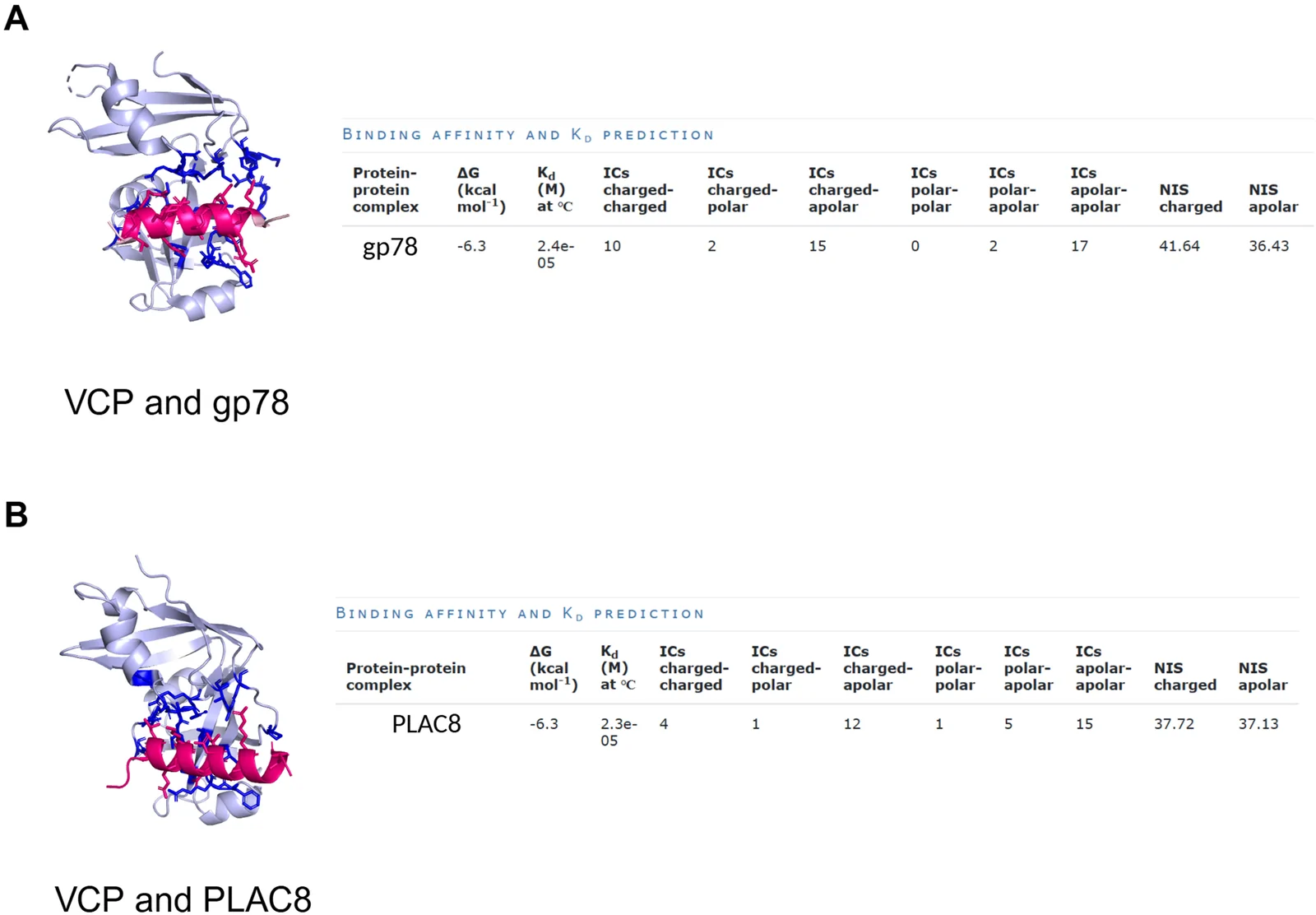
VCP N-terminal domain binds to PLAC8 via a VIM-like interaction. **(A)** Crystal structure of the VCP-interacting motif (VIM) of gp78 C-terminal helix and the corresponding region of the VCP N-terminal domain, with interacting residue sidechains highlighted as sticks. The binding properties determined by the Prodigy webserver^150^ are displayed in the table with energy of binding (ΔG), dissociation constant (K_d_), number of different types of intermolecular contacts (IC) within 5.5 Å, and the percentage of the charged and apolar non-interacting surface (NIS%) of the complex. **(B)** A model of the PLAC8 C-terminal helix (94–115) and VCP N-terminal domain generated by AlphaFold3,^151^ with interacting residues displayed and binding properties outlined the same as in Figure 7A. Protein structure is displayed as cartoons with interacting residue side chains shown as sticks. The VCP N-terminal domain model is colored grey with interacting residues highlighted in blue. gp78 and PLAC8 are colored hot pink. Atomic models are displayed using PyMOL.^149^

The canonical sequence for a VCP-interacting motif (VIM) is R*X*_5_AA*X*_2_R.^28^ There are two potential sequences in PLAC8 which contain this combination of arginines (R), with the most likely contender being the C-terminal helix (residues 94–115).^28^ We have carried out molecular docking experiments and binding analysis of this PLAC8 C-terminal helix to the VCP N-terminal domain (Fig. 7). Single copies of sequences of the VCP N-terminal domain (residues 1–186) and the PLAC8 C-terminal helix (residues 94–115) were input into the AlphaFold server. The best output model of VCP–PLAC8 was input into the Prodigy webserver to predict binding properties of the two molecules, as well as Chains A and C from the crystal structure of VCP N-terminal domain bound to the gp78 C-terminal helix. The C-terminal helices of gp78 and PLAC8 dock into the groove between the two lobes of the VCP N-terminal domain, which is typical for VIMs. The ΔG values for both models are the same at −6.3 kJ/mol, and the K_d_ values for both models are almost identical at 24 μM (gp78) and 23 μM (PLAC8). There is a small variation between the numbers of the different types of intermolecular contacts, but the percentage of the charged and apolar non-interacting surfaces of the complex is very similar. This result provides one mechanistic explanation for the direct binding between PLAC8 and VCP observed in the study by Sun et al, although further *in vitro* studies to confirm this mechanism are required.

The behavior of PLAC8 is very similar to that of another recently identified interactor of VCP, VCP cofactor nuclear family member 1 (VCF1), which is shown to increase polyubiquitinated substrate processing by the ternary complex VCP–Ufd1–Npl4.^104^ Before knowledge of PLAC8's interaction with VCP, it was established that PLAC8 interacted with and upregulated the PI3K/AKT/NFκB pathway, leading to suppressed apoptosis in triple-negative breast cancer cells.^105^ VCP is also known to activate the NFκB pathway by promoting the proteasomal degradation of IκB in a manner similar to wild-type p53. It is now established that PLAC8 activates VCP, and this increases the degradation of wild-type p53. Thus, it seems plausible that the oncogenic effect of PLAC8 in activating the PI3K/AKT/NF-kB may be mediated through interactions with VCP.^105^ A separate study showed that exposure to cadmium in prostate cells resulted in activation of the NFκB pathway. One downstream effect of this exposure was the up-regulated expression of PLAC8.^106^ Thus, cadmium may induce a positive feedback loop in which the NFκB pathway is up-regulated via PLAC8. This leads to further activation through VCP-mediated degradation of the inhibitor of NFκB.

Shifting from proteasomal degradation to autophagy, PLAC8 was shown to have context-dependent effects. In the same study looking at cadmium-exposed prostate cancer, up-regulated PLAC8 disrupted autophagy by preventing fusion of autophagosomes and lysosomes.^106^ As mentioned above, this stage of autophagy is also regulated by VCP, suggesting another possible link between VCP and PLAC8.^106^ In pancreatic cancer, induction of PLAC8 is essential for tumor progression through the activation of autophagy.^107^ In a study by Kinsey et al examining pancreatic ductal adenocarcinoma cell lines, PLAC8 expression levels were up-regulated in response to K-Ras and p53 mutations, both of which are highly prevalent in pancreatic ductal adenocarcinoma and required for disease progression.^107^ They further showed that PLAC8 induction occurred due to a loss of wild-type p53 function rather than as a mutant gain-of-function effect. This corroborates the results of a second study by Yang et al which showed that early in pancreatic cancer, autophagy has a tumor suppressive effect, while later it acts as a driver of tumor progression.^108^ Thus, it seems plausible in this context that loss of wild-type p53 occurs as part of cancer progression, activating autophagy, and this proceeds at least in part through up-regulation of PLAC8. In another study on human trophoblast cells, PLAC8 was shown to increase autophagy by up-regulating transcription of autophagy genes.^109^ Initially, this effect was reversed by wild-type p53, but over time, wild-type p53 was degraded, and this correlated with PLAC8 co-localization. This is likely a further example of PLAC8 inducing VCP-mediated wild-type p53 degradation as seen in non-small cell lung carcinoma,^83^ and this loss of wild-type p53 leads to increased PLAC8-mediated autophagy as seen in pancreatic cancer.^107^ While Kinsey et al showed that in pancreatic ductal adenocarcinoma, up-regulated PLAC8 was due to loss of wild-type p53, a study by Zhang et al comparing metastatic osteosarcomas from *p53*^*R172H/+*^ mice and non-metastatic osteosarcomas from *p53*^*+/−*^ mice showed that PLAC8 was up-regulated by gain-of-function R175H p53 (R172H in mice).^110^ These findings highlight the interplay between and reciprocal regulation of wild-type/mutant p53 and PLAC8, and compound the potential importance of VCP as a mediator of these effects.

### VCP and SVIP

There is a connection between the effects of PLAC8 and another cofactor of VCP, SVIP, which appears to drive opposite effects on glioblastoma progression mediated by VCP. SVIP binds to VCP via its VIM^111^^,^^112^ with up to six SVIP monomers binding to a single VCP hexamer (6:1 ratio).^113^ This ability has been shown to effectively regulate VCP activity by competitively preventing other cofactors from binding.^111^^,^^113^ A recent study by She et al found that PLAC8 drives temozolomide resistance in glioblastoma cells.^114^ Overexpression of PLAC8 in temozolomide-treated glioblastoma cells increased levels of phosphorylated AKT and mTOR, led to p62 accumulation with reduced LC3-II/LC3-I ratio, and decreased cleaved caspase-8 and Bax. Each of these effects was reversed by rapamycin. These findings indicate that PLAC8 promotes temozolomide resistance by activating the AKT/mTOR pathway, suppressing autophagy, and inhibiting apoptosis. The authors also reported the presence of a PLAC8–AKT interaction through co-immunoprecipitation. Since VCP is a known activator of the AKT/mTOR pathway and directly binds to PLAC8 and AKT, the observed PLAC8–AKT interaction and the oncogenic action of PLAC8 in glioblastoma may be mediated by VCP.^115^, ^116^, ^117^ Furthermore, this is another example of the context-dependent effect of PLAC8 on autophagy, in this case showing PLAC8 having an inhibitory effect.

**Figure 8:**
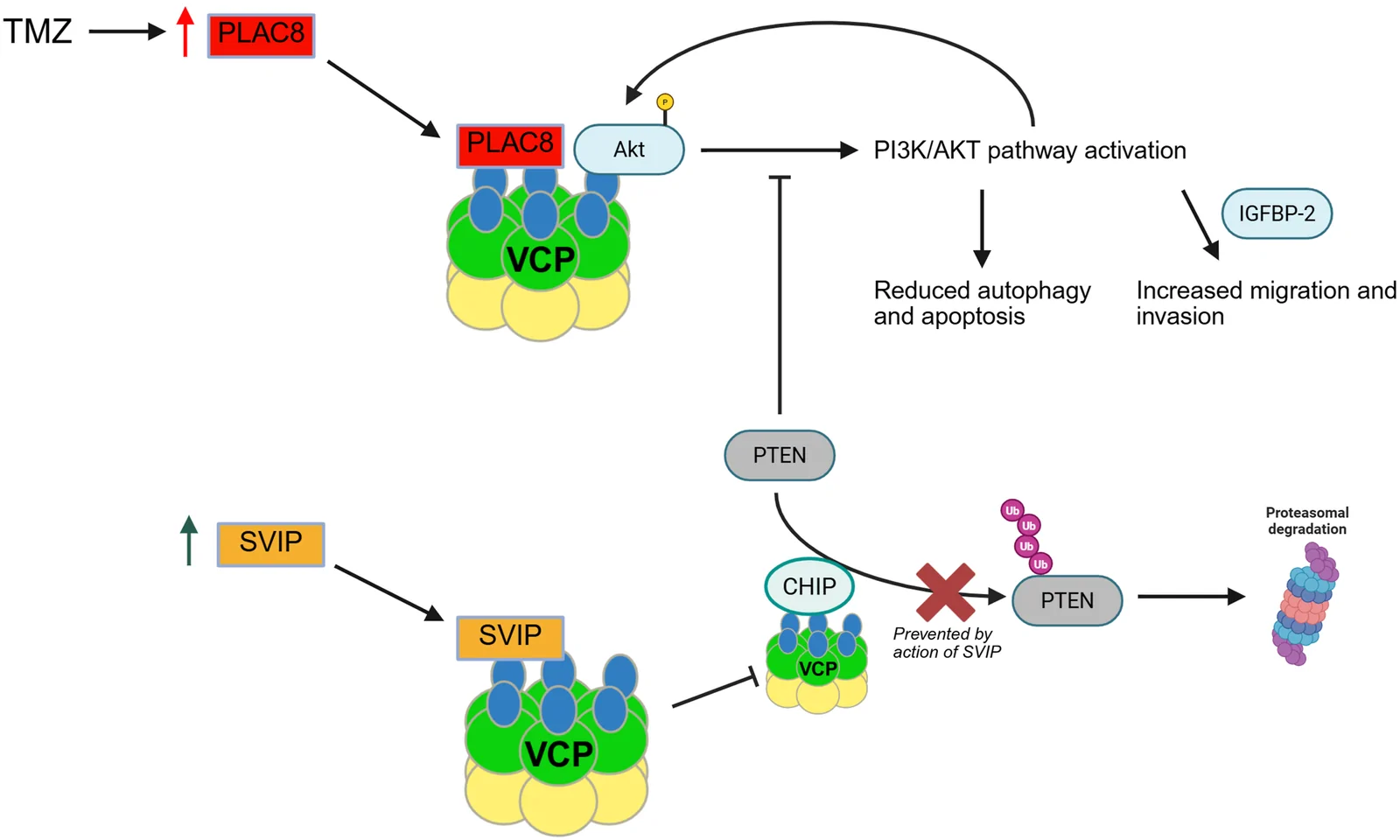
Opposite effects of PLAC8 and SVIP on temozolomide (TMZ) resistance in glioblastoma are mediated by VCP. TMZ treatment increases levels of PLAC8. PLAC8 promotes activation of the PI3K/AKT pathway, likely via combined interaction with VCP and phosphorylated AKT. This leads to reduced autophagy and reduced apoptosis. SVIP outcompetes E3 ubiquitin ligase CHIP from binding to VCP, which stabilizes PTEN by preventing its ubiquitination and proteasomal degradation. PTEN down-regulates the PI3K/AKT pathway, with specific effects on the increased migration and invasion mediated by IGFBP-2

The idea that VCP plays a crucial role in glioblastoma is corroborated by other studies showing that SVIP, an established inhibitor of VCP, has the opposite effect to PLAC8, a VCP activator.^96^ In one such study, increased SVIP levels outcompeted the E3 ubiquitin ligase CHIP for VCP binding, preventing CHIP-mediated ubiquitination of the tumor suppressor protein PTEN. As a result, PTEN was stabilized, and its cellular levels increased. In this context, PTEN down-regulates the PI3K/AKT/mTOR pathway, leading to increased autophagy and reduced levels of insulin-like growth factor binding protein 2 (IGFBP-2), a major factor involved in migration and invasion. Together, these findings suggest that in glioblastoma, VCP regulation of both the PI3K/AKT/mTOR pathway and autophagic flux is controlled in opposite directions by PLAC8 (activation) and SVIP (inhibition) (Fig. 8).

The anti-cancer effect of SVIP has been demonstrated in a study on glioma cells, showing that increased androgen receptor levels reduced SVIP expression, leading to reduced wild-type p53 levels and increased cell proliferation.^118^ The authors attributed this finding to reduced expression of wild-type p53; however, wild-type p53 levels were measured by Western blotting. It is therefore plausible that this effect was due to increased degradation rather than reduced transcription. In a second study looking at SVIP in pancreatic cancer, VCP knockdown reduced migration and invasion in two mutant p53 cell lines (PANC-1, Mia-PACA-2). SVIP knockdown had the opposite effect, indicating that the action of VCP is pro-tumorigenic and SVIP acts to suppress the effects of VCP.^119^ SVIP's effects appear to depend on p53 status. In a breast cancer study, SVIP silencing enhanced proliferation in wild-type p53 cell lines but did not affect proliferation in two mutant p53 cell lines: T47D (L194F) and SK-BR-3 (R175H).^120^ However, SVIP silencing increased migration and invasion in both wild-type p53 and mutant p53 cell lines. Given that SVIP is a VCP inhibitor, one explanation for this phenotype in wild-type p53 cell lines is that SVIP inhibits cell proliferation by reducing VCP-mediated proteasomal degradation of wild-type p53, likely by outcompeting Ufd1–Npl4 cofactors binding to VCP N-terminal domains. The effect is diminished in mutant p53 cell lines, where mutant p53 does not have the same anti-proliferative effect as wild-type p53 and is less affected by VCP-mediated proteasomal degradation. The context-dependent effects of SVIP may also depend on the VCP cofactor displaced; in glioblastoma, SVIP outcompetes CHIP, preventing PTEN ubiquitination, whereas in wild-type p53 breast cancer cells, SVIP outcompetes Ufd1–Npl4. Alternatively, SVIP is also known to redirect VCP towards localization at lysosomal membranes and the plasma membrane, which correlates with increased autophagy and lysosomal membrane stability.^121^^,^^122^ Thus, SVIP may reduce the pool of VCP available for proteasomal degradation by redirecting VCP to these membranes.

The feature of SVIP outcompeting other cofactors for binding to VCP is not isolated to cancer. In cells expressing the Z variant of alpha-1 antitrypsin (A1AT), SVIP overexpression reduces Hrd1-mediated ubiquitination of Z-A1AT, preventing its VCP-mediated exit from the endoplasmic reticulum (ER) and subsequent proteasomal degradation.^123^ Buildup of Z-A1AT leads to increased ER stress, which further elevates SVIP levels, exacerbating the detrimental accumulation of Z-A1AT. A similar effect is observed in cells expressing the CFTRDeltaF508 mutant form of the cystic fibrosis protein, in which SVIP inhibits gp78-mediated ubiquitination of the misfolded protein and reduces its degradation.^124^ A study by Ramzen et al demonstrated that SVIP underwent N-terminal acylation, specifically myristylation at glycine-2 (G2) and palmitoylation at cysteine-4 and cysteine-6 (C4 and C6).^125^ SVIP interaction with VCP was highly dependent on G2 modification and partially dependent on C4/6 modification, with cysteine mutagenesis ablating SVIP's membrane association and its ability to guide VCP localization within cells.^125^ Similarly, acylation inhibitors prevented the same SVIP-mediated effects. Given SVIP's anti-tumorigenic activity, inhibiting SVIP–VCP interactions is unlikely to be a useful therapeutic avenue for cancer, but it may prove viable for treating SVIP-induced pro-fibrotic effects. On the other hand, SVIP overexpression can have a beneficial effect on hepatic fibrotic diseases. Increased SVIP levels outcompete Hrd1 and CHIP binding to VCP, thereby reducing degradation of their substrates Nrf2 and transcription factor EB (TFEB), both of which up-regulate autophagy-associated genes with anti-fibrotic effects.^97^

In the same study by Ramzen et al examining SVIP acylation, it was observed that the interaction of SVIP and the VCP-R155H mutant was highly cytotoxic.^125^ Cell death occurred in a caspase-independent manner and was not prevented by inhibition of proteasomal degradation or autophagy. VCP-R155H is a gain-of-function mutant, demonstrating increased ATPase and unfoldase activity.^126^, ^127^, ^128^ Furthermore, Ramzen et al saw that inhibiting SVIP acylation, thus preventing its ability to guide VCP (WT or R155H mutant) to the lysosomes, reduced cytotoxicity. In an earlier study examining the effects of VCP on lysosomal dynamics, Johnson et al showed that expression of the VCP R155H mutant or VCP knockdown had a similar lysosomal destabilizing effect.^122^^,^^129^ This combination of results suggests that VCP is essential for maintaining lysosomal stability, and that increased VCP activity displayed by the R155H gain-of-function mutant induces instability in the same way the absence of VCP does. Given the role of SVIP in autophagic and lysosomal VCP functions, acylation inhibitors may prove to be viable treatments for multisystem proteinopathy and neurodegenerative disorders, as well as fibrotic diseases.

## Future directions and therapeutic implications

VCP is well established as both a prognostic marker and a therapeutic target in cancer.^53^ Previous reviews have particularly focused on ATPase inhibitors and allosteric or covalent inhibitors binding in the D1-D2 domain region, their progression from preclinical models to early clinical evaluation,^26^ and, most recently, identifying potential for combinatorial therapies with other drug classes such as DNA damage inhibitors and signaling pathway inhibitors.^130^ Key challenges associated with targeting VCP for cancer treatments include off-target effects and dose-limiting toxicity,^131^ as well as the development of resistant mutants.^132^ In this review, we have focused on VCP as a coordinator of proteasomal degradation and autophagy, driven by competitive cofactor binding at the N-terminal domain, leading to context-dependent regulation of key cancer proteins such as p53. Building on this framework, the most immediate future directions are strategies that modulate VCP function more selectively than global ATPase inhibition, either by disrupting the VCP–Ufd1–Npl4 proteasomal degradation complex, biasing VCP toward autophagy, or reprogramming VCP for targeted proteolysis.

### Inhibiting formation of VCP–Ufd1–Npl4 proteasomal degradation complex

A major future direction is to target the VCP–Ufd1–Npl4 axis to reduce VCP-mediated proteasomal degradation while preserving other VCP functions. VCP overexpression is frequently associated with suppression of tumor suppressive proteins and with increased capacity to resolve proteotoxic stress through proteasomal degradation. Inhibiting the formation of the VCP–Ufd1–Npl4 complex, preventing canonical VCP substrate recognition, therefore represents a strategy to inhibit VCP-dependent degradation of selected clients without broadly disabling the ATPase activity required for other VCP-linked pathways.

Disulfiram-derived mechanisms that converge on Npl4 illustrate the feasibility of targeting this axis. Disulfiram and CuET derivatives cause Npl4 aggregation, preventing formation of the ternary complex and inducing cytotoxic effects across multiple cancer types.^133^, ^134^, ^135^ Although disulfiram has pleiotropic effects, these studies support the principle that perturbation of Npl4-dependent VCP function can reduce tumor cell fitness. In parallel, thonzonium bromide has been reported to inhibit the VCP–Npl4 interaction, leading to increased STAT3 signaling and suppression of tumor-infiltrating regulatory T cells. This treatment enhances anti-tumor immunity, indicating that Ufd1–Npl4 axis targeting can target both intrinsic tumor cell proteostasis and the tumor microenvironment.^136^ A separate mechanistic advance is the development of adaptor-specific peptide inhibitors, specifically engineered to disrupt interactions within the VCP–Ufd1–Npl4 ternary complex, preventing substrate unfolding activity.^137^ These peptides establish a strategy to target specific VCP adaptors to prevent their functions while leaving others unaffected, and need not be limited to VCP–Ufd1–Npl4 interactions.

### Strategies to bias VCP towards autophagy rather than UPS

A second future direction is to bias VCP away from UPS routing and toward autophagy. In cancer, this strategy is complex because autophagy can be either tumor suppressive or tumor promoting depending on disease stage and context. However, the concept remains attractive because selectively enhancing autophagic routing may allow degradation of aggregation-prone oncogenic proteins while reducing degradation of tumor suppressors that are preferentially targeted through Ufd1-Npl4-dependent proteasomal pathways. Although the effects on autophagy have not been the focus, studies of nucleotide competitive or allosteric inhibitors targeting D1/D2 VCP domains predominantly indicate autophagy inhibition as well as inhibition of proteasomal degradation.^26^^,^^55^

There is now direct evidence that small molecules can modulate VCP to enhance clearance pathways rather than inhibit them. Work from Wrobel et al showed that compounds activating VCP D1 ATPase enhance clearance of neurotoxic proteins through both autophagic and proteasomal mechanisms,^138^ and that these compounds have differential effects on pathway utilization.^139^ This establishes that VCP can be pharmacologically tuned to uncouple UPS and autophagy outputs. SMER28 is one compound which has been reported to bind VCP/p97, increase macroautophagy flux of aggregated proteins via enhanced PtdIns3K complex I activity, while also increasing proteasomal clearance of misfolded soluble proteins.^138^

Gossypol acetate provides a second example that connects directly to VCP cofactor interactions.^41^ In the context of mutant huntingtin, gossypol was reported to act as a modulator of VCP that promotes the formation of a VCP–LC3–mHTT ternary complex and enhances autophagic degradation of the aggregation-prone substrate. However, gossypol has also been shown to target the Bcl-2 family of proteins,^140^ and therefore its effects in cancer models are likely to reflect both proteostasis-linked and apoptosis-linked mechanisms. For cancer translation, the key future direction is to delineate the VCP-dependent effects and to determine whether the VCP–LC3 recruitment mechanism can be recapitulated by more selective ligands.

The p53 axis provides a strong rationale for exploring this therapeutic direction. VCP has been implicated in suppression of wild type p53 through enhanced proteasomal degradation in multiple settings,^82^^,^^83^ whereas VCP has also been shown to stabilize mutant p53 in pancreatic cancer.^86^ These opposing effects suggest that VCP can either promote degradation or stabilization depending on p53 state and cellular context. This supports a testable model in which Ufd1–Npl4 axis inhibition could protect wild-type p53 from proteasomal turnover, while simultaneously enhancing autophagic routing could penalize oncogenic, stress-stabilized proteins, potentially including subsets of mutant p53. Delineating the role of key E3 ubiquitin ligases and deubiquitinases would provide an alternative avenue for specifically targeting mutant p53 stabilization mediated by VCP.

SVIP provides a complementary route to bias VCP toward autophagy while limiting UPS routing, through competitive cofactor binding at the N-terminal domain. Structural studies have shown that VIM-containing cofactors bind to the N-terminal domain in a mutually exclusive manner, and SVIP can bind to VCP at high stoichiometry, enabling it to outcompete other cofactors.^28^^,^^111^ Functionally, SVIP is associated with relocalization of VCP toward lysosomal membranes and plasma membrane compartments,^121^ and has been implicated in lysosomal stability and autophagosome–lysosome fusion,^122^ with the overall effect of increased autophagy.^97^^,^^121^ These observations support a model in which SVIP reduces the pool of VCP available for Ufd1–Npl4-mediated proteasomal degradation while increasing VCP's engagement in autophagy-linked and lysosome-linked functions.

A key therapeutic implication of SVIP is that it may counteract pro-cancer VCP functions that depend on recruitment of E3 ubiquitin ligases and deubiquitinases. Many of the VCP-dependent cancer mechanisms described in this review and outlined in Figure 2, Figure 3, Figure 4, Figure 5 involve VCP acting as a scaffold to recruit DUBs that stabilize oncogenic factors or to facilitate ubiquitination and degradation of tumor suppressors. SVIP has been shown in other proteostasis contexts to inhibit interactions between VCP and ERAD-associated E3 ligases such as gp78 and Hrd1, and to alter degradation outcomes for misfolded substrates, including Z-A1AT^123^ and CFTRΔF508.^124^ These findings support the broader concept that SVIP can reshape VCP's interaction network with ubiquitin regulators. In cancer, this provides a plausible mechanism by which increased SVIP function could reduce VCP-dependent stabilization of pro-oncogenic proteins, while also stabilizing tumor suppressors that are degraded through VCP-dependent proteasomal pathways. SVIP has been associated with reduced proliferation in wild-type p53 settings, and reduced migration and invasion in multiple contexts, including in cell lines where p53 is mutant.^118^^,^^120^ In glioblastoma, SVIP acts in opposition to PLAC8,^83^^,^^96^ consistent with SVIP restraining VCP-driven activation of PI3K/AKT/mTOR signaling and altering autophagic flux. Together, these data support SVIP as a routing cofactor that can oppose pro-tumor VCP programs.

A final, related therapeutic strategy is subcellular compartmentalization beyond lysosomes. Nuclear import and nucleocytoplasmic shuttling of VCP are regulated by defined cofactors, notably p37 and VCF1/2.^141^^,^^142^ Since nuclear VCP contributes to DNA replication and DNA damage repair, limiting nuclear localization shows significant therapeutic potential, particularly as a radiosensitization strategy 143, 144, 145, 146. Inhibiting nuclear import by disrupting VCP–VCF1/2 interaction through specific peptide inhibitors discussed earlier in the section may be a viable approach.^137^

### VCP-directed PROTAC strategies

A longer-term direction is to reprogram VCP for targeted proteolysis, rather than inhibiting its activity. A recent study described a VCP-directed proteolysis-targeting approach in which a UBX domain module derived from FAF1 was fused to camelid nanobodies to drive selective proteasome-mediated degradation of chosen targets in a ubiquitin-independent manner.^147^ This provides an exciting therapeutic strategy, in which VCP is treated as degradative machinery that can be harnessed to target specific proteins. In principle, VCP-directed degraders could be designed to recruit targets into specific VCP cofactor states, or to couple recruitment with cofactor competition strategies that bias processing outcomes. Although speculative, this raises the possibility that engineered recruiters could be combined with routing cofactors such as SVIP to tune whether recruited substrates are processed through proteasomal degradation or routed through alternative clearance pathways. The key future work required for translation is to define substrate classes that are compatible with VCP-mediated extraction and unfolding, establish specificity, and determine whether VCP can be harnessed for selective degradation without triggering proteostasis-related side effects.

## CRediT authorship contribution statement

**Charlie J. Hitchman:** Writing – review & editing, Writing – original draft, Conceptualization. **Julia I. Phillips:** Writing – review & editing, Writing – original draft. **Hee-Won Park:** Writing – review & editing, Writing – original draft, Conceptualization. **Hua Lu:** Writing – review & editing, Writing – original draft, Funding acquisition, Conceptualization.

## Funding

H. L. was in part supported by 10.13039/100000002National Institutes of Health (No. U01CA252964), the 10.13039/100006153Ladies Leukemia League Foundation, the Krewe de Pink Foundation, and the Reynolds and Ryan Families Endowed Chair, the ACS Discovery Boost grant, and the Louisiana BOR RSC funds.

## Conflict of interests

The authors declared no competing interests.
