# Supplementary material for: VCP at the crossroads of UPS and autophagy in cancer
Source: Genes Dis. 2026 May 28;14(1):102264. doi: 10.1016/j.gendis.2026.102264 (PMC13579142; doi:10.1016/j.gendis.2026.102264)
Supplement: Supplementary file 1 [file mmc1.docx]

**Table S1. Cancer-type comparison of distinct VCP associated tumorigenic mechanisms**

| **Cancer type** | **Primary VCP-linked mechanism node** | **General pathway** | **Phenotype outcome (functional)** | **Clinical association** | **Potential therapeutic strategy** | **Key refs** |
| --- | --- | --- | --- | --- | --- | --- |
| **Bladder** | VCP–MRN retention on chromatin blocks; MRN disassembly at DNA damage sites.  VCP stabilizes Beclin 1 and promotes autophagy by downregulating ataxin‑3.  circHIPK3 inhibits the VCP–Beclin 1 interaction.  CB-5083 (VCP inhibitor) + luteolin synergistically reduce BC viability, induce G1 arrest and apoptosis. | DDR/genome stability  Autophagy regulation (VCP–Beclin 1); DUB axis (ataxin‑3)  Proteostasis stress (ER stress/UPR)  Apoptosis regulation (Bcl-xl/Mcl-1)  Cell-cycle arrest. | Invasiveness; radioresistance.  circHIPK3 downregulation promotes proliferation;  CircHIPK3 overexpression suppresses autophagy and proliferation.  Synergistic CB-5083 + luteolin apoptosis and G1 arrest in vitro; reduced tumor growth in vivo. | Reduced survival post-cystectomy.  CircHIPK3 lower in bladder cancer tissues  Autophagy-related proteins up in tumors vs controls. | Radiozation via targeting the VCP–MRN axis.  Targeting circHIPK3/VCP–Beclin 1 axis to modulate protective autophagy  Luteolin + CB-5083 combination therapy to improve efficacy and reduce CB-5083 dose-limiting toxicity/resistance. | Kilgas et al., 2021; [1]  Wang et al., 2023; [2]  Sun et al., 2025; [3] |
| **Breast** | p21/p27 degradation supports proliferation and therapy resistance.  pSer784-VCP promotes chromatin protein extraction during the DNA damage response.  VCP maintains cancer stem cells; VCP loss triggers UPR and CSC loss.  SVIP (endogenous VCP/ERAD inhibitor) modulates proliferation (p53-dependent) and migration  VCP-enriched sEVs from brain-tropic breast cancer cells deliver VCP to brain endothelium; VCP implicated in VEGFR2 trafficking and BBB dysfunction (brain metastasis axis).  TNBC: VCP–TMEM63A–DERL1 axis; TMEM63A undergoes TOLLIP-mediated autophagic degradation and is stabilized by VCP  VCP inhibition triggers cancer-selective paraptosis; ATF4–DDIT4 suppresses mTORC2/Akt (Akt activity increases vulnerability).  TNBC: VCP promotes proteasomal degradation of NRF3; NRF3–VCP mutual regulation impacts ROS–ERK signaling and migration/invasion. | Cell-cycle control.  DDR/genome stability.  ER stress/UPR–ISR (proteostasis stress).  ERAD regulation (SVIP–VCP; Hrd1–p53 axis).  Autophagy/lysosome regulation  EV trafficking/BBB dysfunction (brain metastasis).  Akt–mTOR signaling; non-apoptotic cell death (paraptosis).  Redox/ROS–ERK signaling | Proliferation; reduced chemotherapy efficacy; CSC maintenance.  Altered migration/invasion with SVIP perturbation in a p53-status dependent manner.  BBB permeability and angiogenesis dysfunction supporting brain metastasis programs.  TNBC progression via VCP-stabilized TMEM63A and autophagy receptor pathway.  Paraptotic death upon VCP inhibition in breast cancer–selective contexts with Akt-linked vulnerability.  NRF3 stabilization upon VCP knockdown increases ROS, decreases p-ERK, and suppresses migration/invasion | Reduced overall survival in breast cancer.  SVIP expression is altered in tumors, with SVIP mRNA higher in primary tumors and SVIP protein reported lower versus normal tissue.  In TNBC, low NRF3 protein is associated with poorer prognosis and discordance between NRF3 mRNA and protein suggests post-translational suppression. | pSer784-VCP as a response biomarker and rationale to combine VCP-axis targeting with DDR-directed therapy.  CSC vulnerability via proteostasis stress on VCP loss or inhibition.  Exploit SVIP–ERAD modulation (SVIP–Hrd1–p53 axis) in subtype- and p53-stratified settings.  Target VCP or EV transfer to limit BBB dysfunction and brain metastasis.  VCP inhibitors as paraptosis inducers with Akt as a predictive biomarker or combination rationale.  In TNBC, inhibit VCP or stabilize NRF3 to suppress migration via the ROS–ERK axis while avoiding ROS scavenging that restores migration. | Zhu et al., 2020; [4]  Shi et al., 2020; [5]  Kartal et al., 2025; [6]  Teles et al., 2024; [7]  Atalay Şahar & Ballar Kirmizibayrak, 2023; [8]  Zhang et al., 2023; [9]  Lee et al., 2024; [10]  Zheng et al., 2024; [11] |
| **Cholangiocarcinoma** | VCP binds BAP1 and promotes its ubiquitination and proteasome-dependent degradation | UPS/proteostasis  Tumor suppressor degradation (BAP1); apoptosis control. | Promotes proliferation and inhibits apoptosis  VCP inhibition suppresses growth and induces apoptosis. | BAP1 downregulation frequent in CCA and associated with poor prognosis | VCP inhibition as strategy to restore BAP1 protein levels and suppress CCA. | Zhang et al., 2025; [12] |
| **Colorectal** | STAT3-linked VCP axis; VCP knockdown reduces STAT3 phosphorylation.  USP11–VCP promotes autophagy-dependent 5-FU resistance.  p97/VCP is overexpressed in CRC and is targeted by newly designed small-molecule p97 inhibitors with anti-proliferative activity.  CB-5083-resistant p97 mutants reduce K48-ubiquitinated protein turnover and dampen CHOP/NF-κB activation; sulforaphane counteracts resistance and synergizes with CB-5083 in parental cells.  Nuclear VCP promotes CRC progression by binding HDAC1 and facilitating its degradation, increasing fatty-acid-oxidation gene expression (e.g., CPT1A).  XAF1 acts as a VCP adaptor promoting metastasis via the RNF114–JUP axis.  SIK2 promotes VCP hyperphosphorylation to enhance extraction of K48-ubiquitinated chromatin proteins during DNA damage response.  VCP inhibition potentiates immune checkpoint therapy in CRC.  TRIM21 alters VCP–UFD1–NPL4 ERAD complex assembly via K27-linked ubiquitination of UFD1, shifting ERAD capacity and UPR sensitivity. | Signaling (STAT3).  Autophagy regulation (USP11–VCP).  UPS/proteostasis and VCP inhibition.  Drug resistance to VCP inhibition with stress-response signaling (CHOP; NF-κB).  Metabolic rewiring (FAO) with epigenetic regulation (HDAC1).  UPS/ubiquitin signaling and adaptor biology.  DDR/genome stability with chromatin extraction (VCP phosphorylation axis).  ERAD/UPS with immune checkpoint synergy.  ERAD complex regulation (VCP–UFD1–NPL4) and UPR/ER stress. | Proliferation and aggressiveness traits with therapy resistance signatures.  5-FU resistance via autophagy-linked mechanisms.  Inhibits proliferation.  Reduced colony formation/proliferation of CB-5083 resistant cells; synergy in parental cells.  Enhanced CRC growth/progression via FAO program activation.  Promotes CRC cell migration and metastasis.  Radioresistance with improved apoptosis/tumor regression when the SIK2–VCP axis is disrupted.  ERAD modulation with altered sensitivity to ER stress-induced apoptosis | Recurrence; reduced DFS/OS.  VCP overexpression is reported in CRC and supported by cohort-style expression analyses.  Higher SIK2 is associated with poorer prognosis  TRIM21 correlations are reported with ERAD/UPR components across cancers. | Target USP11–VCP–autophagy node to reverse 5-FU resistance  STAT3 readouts as mechanistic marker.  Novel p97 inhibitors (lead: compound 10) with in vivo xenograft activity.  Use of sulforaphane to counteract CB-5083 resistance and enhance CB-5083 efficacy. Target nuclear  VCP/HDAC1–FAO axis as metabolic vulnerability.  Targeting XAF1–VCP–RNF114–JUP axis to limit CRC metastasis.  Exploit the SIK2–VCP phosphorylation axis for radiosensitization.  Combine VCP inhibition with immune checkpoint blockade to improve response.  Leverage TRIM21–UFD1 modulation of ERAD to sensitize tumors to ER stress-based strategies. | Fu et al., 2016; [13]  Sun et al., 2021; [14]  Wang et al., 2022; [15]  Huang et al., 2023; [16]  Xia et al., 2024; [17]  Meng et al., 2025; [18]  Qi et al., 2023; [19]  Yuan et al., 2025; [20] |
| **Cutaneous SCC** | VCP inhibition triggers lethal proteotoxic stress engaging ATF6, IRE1α/JNK1, and GCN2/eIF2α  Amino-acid depletion pathways modulate death. | UPR/ISR stress circuitry | Cell death upon VCP knockdown/inhibition; stress-pathway–modulated sensitivity. |  | VCP dependency in cSCC;  Stress-node modifiers (ATF6, IRE1α/JNK, GCN2/eIF2α) to guide drug combinations. | McHugh et al., 2020; [21] |
| **Esophageal** | Combined radiotherapy and VCP inhibition improves prognosis via ER stress/UPR-induced apoptosis. | ER stress signalling/UPR | Combination treatment attenuates cell proliferation, colony formation, and enhances apoptosis | High VCP associates with reduced OS after radiotherapy. | Prognostic/radioresponse biomarker | Luo et al., 2019; [22] |
| **Gastric** | AKT–VCP interaction and increased phospho-VCP with H. pylori supports infected-cell survival.  VCP knockdown sensitizes to TRAIL via CHOP/DR5 upregulation.  Stool proteomics identifies VCP among proteins enriched in DGC mouse-model stool vs controls and in HDGC patient stool vs healthy controls | Signaling (AKT) + PTM.  UPR/apoptosis | Survival of infected cells  TRAIL sensitization with VCP knockdown.  Candidate non-invasive early-detection biomarker panel for DGC/HDGC. | Reduced DFS/OS.  HDGC patients with CDH1 mutations show increased stool VCP vs healthy controls. | Phospho-VCP as signaling marker.  Combine VCP axis with TRAIL via CHOP/DR5.  Translational: stool-based biomarker panel for early detection. | Yu et al., 2013; [23]  Arai et al., 2016; [24]  Ho et al., 2024; [25] |
| **Glioblastoma** | VCP knockdown alters DNA-PK dynamics implicating repair/radiosensitivity.  HDAC6:VCP ratio associates with temozolomide resistance.  AR represses SVIP (VCP inhibitor),  SVIP loss lowers p53 and increases proliferation in p53wt GBM.  Novel inhibitor V8 directly targets VCP and drives VCP aggregation, initiates mitophagy but blocks flux via lysosomal damage | DDR  UPS/Proteostasis/ER stress  AR signaling; ERAD cofactor axis and p53 stability.  Mitophagy/lysosome integrity | Radioresponse biomarker; TMZ resistance association.  p53-dependent proliferation/apoptosis changes with SVIP perturbation.  Mitophagy-flux blockade–linked cytotoxicity; rescue by restoring lysosomal acidification. | Higher VCP with increasing glioma grade.  AR increases and SVIP decreases with WHO grade.  Higher serum testosterone in glioma versus controls | Radiosensitization via DDR node; HDAC6:VCP ratio as treatment-response biomarker.  AR–SVIP–p53 axis supports p53wt-stratified AR targeting.  VCP inhibitors (V8/NMS873) with non-extractable VCP/Ub accumulation as PD readouts.  Lysosomal acidification status as a response modifier to VCP inhibition. | Jiang et al., 2013; [26] Li et al., 2017; [27]  Bao et al., 2017; [28] Cao et al., 2026; [29] |
| **Hematological** | VCP knockdown reduces invasion and induces apoptosis in B-cell lymphoma.  VCP drives tumor exosome secretion (Jurkat).  Treatment response associations (ALL prednisone; MM dependency).  Global/subcellular proteomics after p97/VCP inhibition (CB‑5083) to map compartment-specific protein regulation. VCP elevated in MM; VCP knockdown reduces proliferation via NF-κB pathway.  Novel VCP inhibitor VCP20 suppresses MM proliferation and osteoclast differentiation by inhibiting ubiquitin-dependent degradation of IκBα → NF-κB inactivation; MM-derived exosomal VCP partially alleviates VCP20 effects.  Design and validation of new VCP/p97 (ATPase) inhibitors (e.g., compound V12) for AML. | UPS/proteostasis  ER stress/UPR–ISR  Autophagy/lysosome pathways  Secretory/extracellular vesicle biology (exosomes/EVs).  DDR/genome stability.  NF-κB signaling.  Microenvironment interactions  Hippo–YAP signalin  Apoptosis regulation. | Reduced invasion/apoptosis on VCP knockdown  Compartment-specific protein abundance changes after CB‑5083.  VCP knockdown or inhibiton reduces MM cell proliferation, reduces osteoclast differentiation, improved bone destruction, prolongs survival in MM model mice.  High FAT1: CB‑5083 inhibits proliferation and induces apoptosis; FAT1 knockdown confers relative resistance | Reduced OS/DFS in orbital B-cell lymphoma.  Higher VCP in MM patients; associated with shorter survival (TT2 cohort).  FAT1–prognosis association in patient tissues | VCP dependency in myeloma; exosome output as pharmacodynamic readout.  CB‑5083 mechanism-of-action mapping in AML context; hypothesis generation for combination strategies.  VCP20 as a candidate MM therapeutic targeting VCP/NF-κB and bone disease features.  Novel p97 inhibitor series positioned as improved over CB-5083; AML-focused lead optimization.  FAT1 as biomarker for VCP-inhibitor response; potential YAP/UPR combination strategies.  Compound V12 represents novel drug for AML treatment. | Bosque et al., 2016; [30]  Zhu et al., 2018; [31]  Wei et al., 2023; [32]  Huang et al., 2024; [33]  Wang et al., 2025; [34] |
| **Liver (HCC)** | NEAT1 decreases miR-129-5p leading to increased VCP and growth/migration.  Sorafenib blocks VCP Tyr phosphorylation triggering ER stress and autophagy-dependent death.  PTPRO dephosphorylates VCP to enhance doxorubicin effects.  VCP upregulated; promotes proliferation/migration/invasion via PI3K/AKT/mTOR activation.  VCP interacts with HMGB1 (VCP D1 domain; HMGB1 A-box) and stabilizes HMGB1 by reducing its ubiquitin–proteasome degradation. | ncRNA regulation  PTM (Tyr-P)  UPR  Autophagy  PI3K/AKT/mTOR  UPS-dependent HMGB1 stabilization | Migration/growth; drug-sensitization via VCP Tyr-P state.  Enhanced proliferation, migration, invasion; accelerated tumor growth in vivo (subcutaneous model). | Reduced DFS/OS.  Higher VCP in tumors; associated with advanced TNM stage and poorer prognosis. | VCP phosphorylation state as drug-response lever  Integrate kinase/phosphatase context with chemo.  VCP–HMGB1 axis as target; HMGB1 inhibition attenuates VCP-driven phenotypes. | Fang et al., 2017; [35]  Hsu et al., 2013; [36]  Pu et al., 2022; [37] |
| **Lung (NSCLC)** | VCP is linked to proteasomal regulation of wild-type p53.  miR-129 targets VCP and suppresses migration/invasion/proliferation.  VCP loss is linked to ER stress with EMT/chemoresistance signatures.  KRT80 interacts with VCP and depends on VCP for protein stability, supporting malignant behavior and PI3K/AKT-linked signaling.  PLK1-driven VCP Thr76 phosphorylation promotes centrosome maturation and spindle orientation, with PP1 reversing the phosphorylation at telophase.  FAT1 expression determines sensitivity to CB-5083, with FAT1 loss activating YAP and dampening CB-5083-induced UPR/ER stress. | UPS/p53 regulation.  miRNA regulation.  UPR/ER stress and EMT coupling.  PI3K/AKT signalling  Mitosis/centrosome control and genome stability.  Hippo–YAP signaling  UPR-mediated response to VCP inhibition. | Reduced migration/invasion/proliferation with miR-129–VCP suppression.  Stress/EMT programs linked to reduced therapy responsiveness in relevant contexts.  KRT80 supports viability and migration, and can mitigate growth inhibition from VCP knockdown in reported models.  Centrosome/spindle defects with chromosome segregation consequences and tumorigenesis linkage in vivo.  High FAT1 associates with CB-5083 sensitivity, while FAT1 loss confers relative resistance. | Reduced DFS/OS is reported in association with implicated VCP-linked axes.  High KRT80 correlates with poorer clinical outcomes.  Thr76-phospho-VCP is reported in lung cancer sections alongside functional assays.  FAT1 is a response determinant with reported prognosis association. | Use the miR-129–VCP axis and stress/EMT signatures for stratification and targeting logic.  Use KRT80 as a diagnostic/prognostic marker and as a VCP-linked dependency candidate  Use Thr76 phospho-VCP as a biomarker and consider PLK1/PP1–VCP axis targeting.  Use FAT1 as a biomarker for VCP-inhibitor response and consider YAP/UPR-informed combination strategies. | Xiao et al., 2015; [38] Huang et al., 2024; [39] Zhu et al., 2022; [40] Liu et al., 2025; [41] |
| **Ovarian (HGSOC)** | VCP essential gene in ovarian cancer  Increased VCP in CCNE1-overexpressing, cisplatin-resistant cells.  VCP inhibitors induce cytotoxicity; synergistic interaction between VCP inhibitors (e.g., CB-5083) and mifepristone. | Proteostasis vulnerability  UPR/ER stress  Apoptosis. | Chemoresistance-linked context (cisplatin resistance).  Synergistic cytotoxicity in ovarian cancer cell lines. | VCP identified as essential gene in ovarian cancer | Candidate vulnerability in CCNE1-high/platinum-resistant disease; inhibitor rationale via essentiality.  Drug synergy strategy: combine VCP inhibitors with UPR-modulating agents (mifepristone) to enhance efficacy. | Cheung et al., 2011; [42]  Bastola et al., 2016; [43]  Bastola et al., 2022; [44] |
| **Osteosarcoma** | NF-κB axis promotes metastasis; anoikis resistance/autophagy link.  Aurora-B increases phospho-VCP to activate NF-κB.  SAP binds VCP relieving anti-apoptotic effect.  miR-129-5p decreases VCP to reduce malignancy.  Dithiocarbamate–copper complexes induce aggregation of NPL4 (p97/VCP cofactor), impairing the VCP/NPL4 axis within the ubiquitin–proteasome system.  VCP recruits USP2 to deubiquitinate/stabilize FASN, preventing its degradation and promoting OS progression | NF-κB signaling  PTM (Aurora-B)  Autophagy  miRNA regulation  UPS/proteostasis; p97/VCP cofactor (NPL4) dysfunction; proteotoxic stress; HSR/UPR  Ubiquitin signaling; lipid metabolism (FASN);  Autophagy | Metastasis; survival/apoptosis regulation.  Proteotoxic stress with heat-shock and unfolded-protein responses; cancer cell death.  Enhanced OS progression and association with poorer outcomes | Reduced malignancy when VCP suppressed. | Target Aurora-B–phospho-VCP–NF-κB cascade; leverage NF-κB/autophagy coupling for combinations.  Repurposing/derivatization of disulfiram–Cu chemistry; NPL4 aggregation as an anticancer strategy.  Target VCP–USP2–FASN axis (and/or FASN) as therapeutic strategy | He et al., 2015; [45]  Wang et al., 2024; [46]  Loffelmann et al., 2023; [47] |
| **Pancreatic (PDAC/PENs)** | miR-198 targets VCP, reducing metastasis and tumor growth.  Nanoparticle-delivered miR-198 sensitizes pancreatic cancer to gemcitabine by downregulating VCP-mediated autophagy.  Lipid droplet–associated VCP is linked to survival biology.  Genetic VCP suppression or pharmacologic VCP inhibition (CB-5083) disrupts proteostasis, increases ER stress, and induces DNA damage, with reported synergy when autophagy is inhibited (chloroquine).  A TME-responsive nanoformulation combines CB-5083 with immune-modulatory components to couple p97 suppression with immunogenic cell death and immune reinvigoration.  VCP promotes invasion/migration, while SVIP shows opposing effects in invasion/migration assays. | Autophagy regulation and chemotherapy response.  Lipid droplet/proteostasis biology.  UPS/proteostasis,  ER stress/UPR  DDR  Proteostasis/UPR linked to tumor immunology in combination nano-therapeutics.  ERAD control of invasive phenotypes (SVIP–VCP axis). | Reduced metastasis and tumor suppression with miR-198–VCP suppression.  Gemcitabine sensitization via disruption of VCP-linked autophagy.  Growth suppression in PDAC models with reported synergy for CB-5083 plus chloroquine.  Immune remodeling and tumor control in reported in vivo combination nano-therapy studies.  Reduced invasion/migration with VCP knockdown and increased invasion/migration with SVIP knockdown in reported assays. | High VCP is reported as prognostically unfavorable in pancreatic cancer contexts. | miR-198–VCP axis as suppressive lever;  Explore lipid droplet–VCP biology as distinct hook. CB-5083 (VCP inhibitor) + chloroquine combination  VCP as DDR/proteostasis node vulnerability in KRAS-mutant PDAC. Combination strategy via nanoformulation: CB-5083 + miR-142 + PD-L1 inhibitor + R848.. VCP as pro-invasive node; SVIP potentially tumor-suppressive regulator of VCP/ERAD | Marin-Muller et al., 2013; [48] Marin-Muller et al., 2023; [49] Lee et al., 2023; [50] Lo et al., 2024; [51] Alimoğulları & Çaylı, 2024; [52] |
| **Prostate** | IL-6/Pim-1-induced VCP drives migration/invasion.  VCP overexpression reduces apoptosis; inhibition triggers ER-stress apoptosis.  Starvation: perinuclear VCP aggregates with reduced mitochondrial ROS.  VCP/p97 promotes neuroendocrine differentiation via Pim1→c‑Myc signaling and induction of autophagy; IL‑6 induces VCP; VCP inhibition (NMS‑873) blocks NED | Inflammatory signaling (IL-6/Pim-1);  UPR/stress adaptation  Autophagy/lysosome;  Cytokine (IL‑6) signaling | Invasion; proliferation; apoptosis resistance; stress adaptation.  Induces NE markers (NSE, CHR‑A) and NED morphology; inhibition of VCP/autophagy arrests NED | Poor prognosis with high VCP. | ER-stress apoptosis upon VCP inhibition; IL-6/Pim-1 as upstream stratifier.  VCP inhibition to block NED/NEPC progression; Pim1/autophagy as co-targets | Duscharla et al., 2018; [53]  Sruthi et al., 2025; [54] |

**Abbreviations:** AKT – protein kinase B; ALL – acute lymphoblastic leukemia; AML – acute myeloid leukemia; Apaf-1 – apoptotic protease activating factor 1; AR – androgen receptor; ATF4 – activating transcription factor 4; ATF6 – activating transcription factor 6; BAP1 – BRCA1 associated protein 1; BBB – blood–brain barrier; Bcl-xL – B-cell lymphoma-extra large; BC – bladder cancer; CCA – cholangiocarcinoma; CCNE1 – cyclin E1; CDH1 – cadherin 1 (E-cadherin); CETSA – cellular thermal shift assay; CHOP – C/EBP homologous protein (DDIT3); CHR-A – chromogranin A; CPT1A – carnitine palmitoyltransferase 1A; CRC – colorectal cancer; cSCC – cutaneous squamous cell carcinoma; circHIPK3 – circular RNA from HIPK3; DDR – DNA damage response; DDIT4 – DNA damage inducible transcript 4; DERL1 – derlin 1; DFS – disease-free survival; DGC – diffuse-type gastric cancer; DOX – doxorubicin; DR5 – death receptor 5; DUB – deubiquitinase; eIF2α – eukaryotic initiation factor 2 alpha; EMT – epithelial–mesenchymal transition; ER – endoplasmic reticulum; ERAD – endoplasmic reticulum–associated degradation; ERK – extracellular signal–regulated kinase; EV – extracellular vesicle; FAO – fatty acid oxidation; FASN – fatty acid synthase; FAT1 – FAT atypical cadherin 1; GBM – glioblastoma; G1 – Growth 1 phase; GCN2 – general control nonderepressible 2; GRP78 – glucose-regulated protein 78; H. pylori – Helicobacter pylori; HCC – hepatocellular carcinoma; HDAC1 – histone deacetylase 1; HDAC6 – histone deacetylase 6; HDGC – hereditary diffuse gastric cancer; HMGB1 – high mobility group box 1; HSR – heat-shock response; IκBα – inhibitor of κB alpha; IRE1α – inositol-requiring enzyme 1 alpha (ERN1); ISR – integrated stress response; ITC – isothermal titration calorimetry; JNK1 – c-Jun N-terminal kinase 1 (MAPK8); JUP – junction plakoglobin; K27 – Lys27-linked ubiquitin chains; K48 – Lys48-linked ubiquitin chains; KRAS – Kirsten rat sarcoma viral oncogene homolog; KRT80 – keratin 80; LC3 – microtubule-associated proteins 1A/1B light chain 3; LARC – locally advanced rectal cancer; miR – microRNA; Mcl-1 – myeloid cell leukemia 1; MDV3100 – enzalutamide; MM – multiple myeloma; MRN – MRE11–RAD50–NBS1 complex; mTOR – mechanistic target of rapamycin; mTORC2 – mechanistic target of rapamycin complex 2; NAC – N-acetylcysteine; NED – neuroendocrine differentiation; NEAT1 – nuclear paraspeckle assembly transcript 1; NEPC – neuroendocrine prostate cancer; NF-κB – nuclear factor kappa B; NRF3 – nuclear factor erythroid 2-related factor 3; NMS-873 – p97/VCP inhibitor NMS-873; NPL4 – nuclear protein localization protein 4; NSE – neuron-specific enolase; NSCLC – non-small cell lung cancer; OS – overall survival; PD-1 – programmed cell death protein 1; PD-L1 – programmed death-ligand 1; PDAC – pancreatic ductal adenocarcinoma; PENs – pancreatic endocrine neoplasms; PI3K – phosphoinositide 3-kinase; PLK1 – polo-like kinase 1; p53wt – wild-type p53; p-ERK – phosphorylated ERK; pSer784 – phosphorylated Ser784; PTM – post-translational modification; PTPRO – protein tyrosine phosphatase receptor type O; R848 – resiquimod; RNF114 – ring finger protein 114; ROS – reactive oxygen species; sEV – small extracellular vesicles; SIK2 – salt-inducible kinase 2; STAT3 – signal transducer and activator of transcription 3; SVIP – small VCP-interacting protein; TME – tumor microenvironment; TMEM63A – transmembrane protein 63A; TMZ – temozolomide; TNBC – triple-negative breast cancer; TNM – tumor–node–metastasis; TOLLIP – toll-interacting protein; TRAIL – TNF-related apoptosis-inducing ligand; TRIM21 – tripartite motif-containing 21; Tyr-P – tyrosine phosphorylation; Ub – ubiquitin; UFD1 – ubiquitin fusion degradation protein 1; UPR – unfolded protein response; UPS – ubiquitin–proteasome system; USP2 – ubiquitin-specific peptidase 2; USP11 – ubiquitin-specific peptidase 11; V12 – VCP inhibitor “V12” (compound name); V8 – VCP-targeting compound “V8” (compound name); VCP – valosin-containing protein; VCP20 – VCP inhibitor “VCP20” (compound name); VEGFR2 – vascular endothelial growth factor receptor 2; XAF1 – XIAP-associated factor 1; YAP – Yes-associated protein.

**Supplementary References**

1.	Kilgas S, Singh AN, Paillas S, Then C-K, Torrecilla I, Nicholson J, Browning L, Vendrell I, Konietzny R, Kessler BM *et al*: **p97/VCP inhibition causes excessive MRE11-dependent DNA end resection promoting cell killing after ionizing radiation**. *Cell Reports* 2021, **35**(8).10.1016/j.celrep.2021.109153

2.	Wang C, Liu T, Wang J, Cheng C, Zhang Z, Zhang J, Huang H, Li Y: **CircHIPK3 negatively regulates autophagy by blocking VCP binding to the Beclin 1 complex in bladder cancer**. *Discover Oncology* 2023, **14**(1):86.10.1007/s12672-023-00689-0

3.	Sun S, Liu P, Xie G, Zheng J: **CB-5083 and luteolin synergistically induce the apoptosis of bladder cancer cells via multiple mechanisms**. *Toxicology and Applied Pharmacology* 2025, **499**:117333.<https://doi.org/10.1016/j.taap.2025.117333>

4.	Zhu C, Rogers A, Asleh K, Won J, Gao D, Leung S, Li S, Vij KR, Zhu J, Held JM *et al*: **Phospho-Ser784-VCP Is Required for DNA Damage Response and Is Associated with Poor Prognosis of Chemotherapy-Treated Breast Cancer**. *Cell Reports* 2020, **31**(10):107745.<https://doi.org/10.1016/j.celrep.2020.107745>

5.	Shi X, Zhu K, Ye Z, Yue J: **VCP/p97 targets the nuclear export and degradation of p27Kip1 during G1 to S phase transition**. *The FASEB Journal* 2020, **34**(4):5193-5207.<https://doi.org/10.1096/fj.201901506R>

6.	Kartal B, Alimoğulları E, Çaylı S: **Investigation of the effects of small VCP-interacting protein (SVIP)on the migration and invasion of breast cancer cells**. *Pathology - Research and Practice* 2025, **269**:155929.<https://doi.org/10.1016/j.prp.2025.155929>

7.	Teles RHG, Villarinho NJ, Yamagata AS, Hiroki CT, de Oliveira MC, Terçarioli GR, Jaeger RG, Meybohm P, Burek M, Freitas VM: **Valosin-containing protein (VCP), a component of tumor-derived extracellular vesicles, impairs the barrier integrity of brain microvascular endothelial cells**. *BBA Advances* 2025, **7**:100130.<https://doi.org/10.1016/j.bbadva.2024.100130>

8.	Atalay Şahar E, Ballar Kirmizibayrak P: **Differential Expression and Function of SVIP in Breast Cancer Cell Lines and In Silico Analysis of Its Expression and Prognostic Potential in Human Breast Cancer**. In: *Cells.* vol. 12; 2023.10.3390/cells12101362

9.	Zhang T-M, Liao L, Yang S-Y, Huang M-Y, Zhang Y-L, Deng L, Hu S-Y, Yang F, Zhang F-L, Shao Z-M *et al*: **TOLLIP-mediated autophagic degradation pathway links the VCP-TMEM63A-DERL1 signaling axis to triple-negative breast cancer progression**. *Autophagy* 2023, **19**(3):805-821.10.1080/15548627.2022.2103992

10.	Lee DM, Kim IY, Lee HJ, Seo MJ, Cho M-Y, Lee HI, Yoon G, Ji J-H, Park SS, Jeong S-Y *et al*: **Akt enhances the vulnerability of cancer cells to VCP/p97 inhibition-mediated paraptosis**. *Cell Death & Disease* 2024, **15**(1):48.10.1038/s41419-024-06434-x

11.	Zheng C, Pan Y, Lin B, Li J, Chen Q, Zheng Z: **NRF3 suppresses the metastasis of triple-negative breast cancer cells by inhibiting ERK activation in a ROS-dependent manner**. *Histol Histopathol* 2024:18786.10.14670/HH-18-786

12.	Zhang P, Liu X, Liu Y, Zhu H, Zheng C, Ling Q, Yan F, He Q, Zhu H, Yuan T *et al*: **VCP Promotes Cholangiocarcinoma Development by Mediating BAP1 Ubiquitination-Dependent Degradation**. *Cancer Science* 2025, **116**(6):1579-1591.<https://doi.org/10.1111/cas.70061>

13.	Fu Q, Jiang Y, Zhang D, Liu X, Guo J, Zhao J: **Valosin-containing protein (VCP) promotes the growth, invasion, and metastasis of colorectal cancer through activation of STAT3 signaling**. *Molecular and Cellular Biochemistry* 2016, **418**(1):189-198.10.1007/s11010-016-2746-6

14.	Sun H, Wang R, Liu Y, Mei H, Liu X, Peng Z: **USP11 induce resistance to 5-Fluorouracil in Colorectal Cancer through activating autophagy by stabilizing VCP**. *Journal of Cancer* 2021, **12**(8):2317-2325.10.7150/jca.52158

15.	Wang F, Li S, Rosencrans WM, Cheng K-W, Stott GM, Mroczkowski B, Chou T-F: **Sulforaphane is Synergistic with CB-5083 and Inhibits Colony Formation of CB-5083-Resistant HCT116 Cells**. *ChemMedChem* 2022, **17**(11):e202200030.<https://doi.org/10.1002/cmdc.202200030>

16.	Huang Y, Wang F, Lin X, Li Q, Lu Y, Zhang J, Shen X, Tan J, Qin Z, Chen J *et al*: **Nuclear VCP drives colorectal cancer progression by promoting fatty acid oxidation**. *Proceedings of the National Academy of Sciences* 2023, **120**(41):e2221653120.10.1073/pnas.2221653120

17.	Xia J, Ma N, Shi Q, Liu Q-C, Zhang W, Cao H-J, Wang Y-K, Zheng Q-W, Ni Q-Z, Xu S *et al*: **XAF1 promotes colorectal cancer metastasis via VCP–RNF114–JUP axis**. *Journal of Cell Biology* 2023, **223**(2):e202303015.10.1083/jcb.202303015

18.	Meng Y, Li S, Lu D-S, Chen X, Li L, Duan Y-f, Wang G-y, Huang W, Liu R-y: **Salt-inducible kinase 2 confers radioresistance in colorectal cancer by facilitating homologous recombination repair**. *MedComm* 2025, **6**(2):e70083.<https://doi.org/10.1002/mco2.70083>

19.	Wang F, Qi Q, Qin B, Wang Y, Huang Y, Li Q, Shen X, Wang X, Yang S, Pan G *et al*: **Targeting VCP potentiates immune checkpoint therapy for colorectal cancer**. *Cell Reports* 2023, **42**(11).10.1016/j.celrep.2023.113318

20.	Yuan C, Liao Y, Si W, Huang M, Li D, Wang F, Quan Y, Yu X, Liao S: **Trim21 modulates endoplasmic reticulum-associated degradation and sensitizes cancer cells to ER stress-induced apoptosis by inhibiting VCP/Npl4/UFD1 assembly**. *Biochimica et Biophysica Acta (BBA) - Molecular Basis of Disease* 2025, **1871**(1):167533.<https://doi.org/10.1016/j.bbadis.2024.167533>

21.	McHugh A, Fernandes K, Chinner N, Ibrahim AFM, Garg AK, Boag G, Hepburn LA, Proby CM, Leigh IM, Saville MK: **The Identification of Potential Therapeutic Targets for Cutaneous Squamous Cell Carcinoma**. *Journal of Investigative Dermatology* 2020, **140**(6):1154-1165.e1155.<https://doi.org/10.1016/j.jid.2019.09.024>

22.	Luo H, Song H, Mao R, Gao Q, Feng Z, Wang N, Song S, Jiao R, Ni P, Ge H: **Targeting valosin-containing protein enhances the efficacy of radiation therapy in esophageal squamous cell carcinoma**. *Cancer Science* 2019, **110**(11):3464-3475.<https://doi.org/10.1111/cas.14184>

23.	Yu C-C, Yang J-C, Chang Y-C, Chuang J-G, Lin C-W, Wu M-S, Chow L-P: **VCP Phosphorylation-Dependent Interaction Partners Prevent Apoptosis in Helicobacter pylori-Infected Gastric Epithelial Cells**. *PLOS ONE* 2013, **8**(1):e55724.10.1371/journal.pone.0055724

24.	Arai MA, Taguchi S, Komatsuzaki K, Uchiyama K, Masuda A, Sampei M, Satoh M, Kado S, Ishibashi M: **Valosin-containing Protein is a Target of 5′-l Fuligocandin B and Enhances TRAIL Resistance in Cancer Cells**. *ChemistryOpen* 2016, **5**(6):574-579.<https://doi.org/10.1002/open.201600081>

25.	Ho C-LC, Gilbert MB, Urtecho G, Lee H, Drew DA, Klempner SJ, Cho JS, Ryan TJ, Rustgi N, Lee H *et al*: **Stool Protein Mass Spectrometry Identifies Biomarkers for Early Detection of Diffuse-type Gastric Cancer**. *Cancer Prevention Research* 2024, **17**(8):361-376.10.1158/1940-6207.CAPR-23-0449

26.	Jiang N, Shen Y, Fei X, Sheng K, Sun P, Qiu Y, Larner J, Cao L, Kong X, Mi J: **Valosin-containing protein regulates the proteasome-mediated degradation of DNA-PKcs in glioma cells**. *Cell Death & Disease* 2013, **4**(5):e647-e647.10.1038/cddis.2013.171

27.	Li Z-y, Zhang C, Zhang Y, Chen L, Chen B-d, Li Q-z, Zhang X-j, Li W-p: **A novel HDAC6 inhibitor Tubastatin A: Controls HDAC6-p97/VCP-mediated ubiquitination-autophagy turnover and reverses Temozolomide-induced ER stress-tolerance in GBM cells**. *Cancer Letters* 2017, **391**:89-99.<https://doi.org/10.1016/j.canlet.2017.01.025>

28.	Bao D, Cheng C, Lan X, Xing R, Chen Z, Zhao H, Sun J, Wang Y, Niu C, Zhang B *et al*: **Regulation of p53 wt glioma cell proliferation by androgen receptor-mediated inhibition of small VCP/p97-interacting protein expression**. *Oncotarget* 2017, **8**(14)

29.	Cao X, Li Y, Guo B, Liu Y, Wang B, Wang H, Lu J, Wei L, Gao Y, Guo Y *et al*: **Targeting VCP with V8 suppresses glioblastoma development via formation of aggregates and disruption of mitophagy flux**. *Cellular Oncology* 2026, **49**(1):21.10.1007/s13402-025-01149-3

30.	Bosque A, Dietz L, Gallego-Lleyda A, Sanclemente M, Iturralde M, Naval J, Alava MA, Martínez-Lostao L, Thierse H-J, Anel A: **Comparative proteomics of exosomes secreted by tumoral Jurkat T cells and normal human T cell blasts unravels a potential tumorigenic role for valosin-containing protein**. *Oncotarget; Vol 7, No 20* 2016

31.	Zhu W, Li D, Xiao L: **Upregulation of valosin-containing protein (VCP) is associated with poor prognosis and promotes tumor progression of orbital B-cell lymphoma**. *OncoTargets and Therapy* 2018, **12**(null):243-253.10.2147/OTT.S182118

32.	Wei R, Cao Y, Wu H, Liu X, Jiang M, Luo X, Deng Z, Wang Z, Ke M, Zhu Y *et al*: **Inhibition of VCP modulates NF-κB signaling pathway to suppress multiple myeloma cell proliferation and osteoclast differentiation**. *Aging* 2023, **15**(16):8220-8236.10.18632/aging.204965

33.	Huang W, Qiu Y, Huynh D, Wang T-Y, Chou T-F: **Proteomics analysis reveals the differential impact of the p97 inhibitor CB-5083 on protein levels in various cellular compartments of the HL-60 cell line**. *microPublication Biology* 2024, **2024**.10.17912/MICROPUB.BIOLOGY.001372

34.	Xueyuan Wang ZL, Tiantian Wen, Hang Miao, Xinran Ye, Meng Lei, Yongqiang Zhu: **Design and Synthesis of Novel 5,6,7,8-Tetrahydropyrido[2,3-D]pyrimidine Derivatives as VCP/p97 Inhibitors for the Treatment of Acute Myeloid Leukemia (AML)**. *Drug Design, Development and Therapy* 2025, **19**:4457-4479. <https://doi.org/10.2147/DDDT.S509036>

35.	Fang L, Sun J, Pan Z, Song Y, Zhong L, Zhang Y, Liu Y, Zheng X, Huang P: **Long non-coding RNA NEAT1 promotes hepatocellular carcinoma cell proliferation through the regulation of miR-129-5p-VCP-IκB**. *American Journal of Physiology-Gastrointestinal and Liver Physiology* 2017, **313**(2):G150-G156.10.1152/ajpgi.00426.2016

36.	Hsu S-h, Motiwala T, Roy S, Claus R, Mustafa M, Plass C, Freitas MA, Ghoshal K, Jacob ST: **Methylation of the PTPRO gene in human hepatocellular carcinoma and identification of VCP as its substrate**. *Journal of Cellular Biochemistry* 2013, **114**(8):1810-1818.<https://doi.org/10.1002/jcb.24525>

37.	Pu Z, Duda DG, Zhu Y, Pei S, Wang X, Huang Y, Yi P, Huang Z, Peng F, Hu X *et al*: **VCP interaction with HMGB1 promotes hepatocellular carcinoma progression by activating the PI3K/AKT/mTOR pathway**. *Journal of Translational Medicine* 2022, **20**(1):212.10.1186/s12967-022-03416-5

38.	Xiao Y, Li X, Wang H, Wen R, He J, Tang J: **Epigenetic regulation of miR-129-2 and its effects on the proliferation and invasion in lung cancer cells**. *Journal of Cellular and Molecular Medicine* 2015, **19**(9):2172-2180.<https://doi.org/10.1111/jcmm.12597>

39.	Huang S, Tong W, Yang B, Ma L, Zhang J, Wang C, Xu L, Mei J: **KRT80 Promotes Lung Adenocarcinoma Progression and Serves as a Substrate for VCP**. *Journal of Cancer* 2024, **15**(8):2229-2244.10.7150/jca.91753

40.	Zhu K, Cai Y, Si X, Ye Z, Gao Y, Liu C, Wang R, Ma Z, Zhu H, Zhang L *et al*: **The phosphorylation and dephosphorylation switch of VCP/p97 regulates the architecture of centrosome and spindle**. *Cell Death & Differentiation* 2022, **29**(10):2070-2088.10.1038/s41418-022-01000-4

41.	Liu X, Zhang P, Ge F, Yuan T, Guo R, Yang S, Yang B, Zhu H, Yan F, He Q: **FAT1 Enhances the Sensitivity of Non-Small Cell Lung Cancer to VCP Inhibitors by Regulating the Activation of the Endoplasmic Reticulum Stress Pathway**. *The FASEB Journal* 2025, **39**(9):e70585.<https://doi.org/10.1096/fj.202500097RR>

42.	Cheung HW, Cowley GS, Weir BA, Boehm JS, Rusin S, Scott JA, East A, Ali LD, Lizotte PH, Wong TC *et al*: **Systematic investigation of genetic vulnerabilities across cancer cell lines reveals lineage-specific dependencies in ovarian cancer**. *Proceedings of the National Academy of Sciences* 2011, **108**(30):12372-12377.10.1073/pnas.1109363108

43.	Bastola P, Neums L, Schoenen FJ, Chien J: **VCP inhibitors induce endoplasmic reticulum stress, cause cell cycle arrest, trigger caspase-mediated cell death and synergistically kill ovarian cancer cells in combination with Salubrinal**. *Molecular Oncology* 2016, **10**(10):1559-1574.<https://doi.org/10.1016/j.molonc.2016.09.005>

44.	Bastola P, Leiserowitz GS, Chien J: **Multiple Components of Protein Homeostasis Pathway Can Be Targeted to Produce Drug Synergies with VCP Inhibitors in Ovarian Cancer**. In: *Cancers.* vol. 14; 2022: 2949.10.3390/cancers14122949

45.	He JY, Xi WH, Zhu LB, Long XH, Chen XY, Liu JM, Luo QF, Zhu XP, Liu ZL: **Knockdown of Aurora-B alters osteosarcoma cell malignant phenotype via decreasing phosphorylation of VCP and NF-κB signaling**. *Tumor Biology* 2015, **36**(5):3895-3902.10.1007/s13277-014-3032-4

46.	Wang S, Nie J, Jiang H, Li A, Zhong N, Tong W, Yao G, Jiang A, Xie X, Zhong Y *et al*: **VCP enhances autophagy-related osteosarcoma progression by recruiting USP2 to inhibit ubiquitination and degradation of FASN**. *Cell Death & Disease* 2024, **15**(11):788.10.1038/s41419-024-07168-6

47.	Loffelmann M, Škrott Z, Majera D, Štarha P, Kryštof V, Mistrík M: **Identification of novel dithiocarbamate-copper complexes targeting p97/NPL4 pathway in cancer cells**. *European Journal of Medicinal Chemistry* 2023, **261**:115790.<https://doi.org/10.1016/j.ejmech.2023.115790>

48.	Marin-Muller C, Li D, Bharadwaj U, Li M, Chen C, Hodges SE, Fisher WE, Mo Q, Hung M-C, Yao Q: **A Tumorigenic Factor Interactome Connected through Tumor Suppressor MicroRNA-198 in Human Pancreatic Cancer**. *Clinical Cancer Research* 2013, **19**(21):5901-5913.10.1158/1078-0432.CCR-12-3776

49.	Marin-Muller C, Li D, Lü J-M, Liang Z, Vega-Martínez O, Crawford SE, Estes MK, Fisher WE, Chen C, Yao Q: **Nanoparticle-Mediated Therapy with miR-198 Sensitizes Pancreatic Cancer to Gemcitabine Treatment through Downregulation of VCP-Mediated Autophagy**. In: *Pharmaceutics.* vol. 15; 2023: 2038.10.3390/pharmaceutics15082038

50.	Lee YS, Klomp JE, Stalnecker CA, Goodwin CM, Gao Y, Droby GN, Vaziri C, Bryant KL, Der CJ, Cox AD: **VCP/p97, a pleiotropic protein regulator of the DNA damage response and proteostasis, is a potential therapeutic target in KRAS-mutant pancreatic cancer**. *Genes Cancer* 2023, **14**:30-49.10.18632/genesandcancer.231

51.	Lo Y-L, Li C-Y, Chou T-F, Yang C-P, Wu L-L, Chen C-J, Chang Y-H: **Exploring in vivo combinatorial chemo-immunotherapy: Addressing p97 suppression and immune reinvigoration in pancreatic cancer with tumor microenvironment-responsive nanoformulation**. *Biomedicine & Pharmacotherapy* 2024, **175**:116660.<https://doi.org/10.1016/j.biopha.2024.116660>

52.	Alimoğullari E, Çayli S: **The function of p97/valosin-containing protein (VCP) and small VCP-interacting protein (SVIP) in invasion and migration of pancreatic cancer cells**. *Turk J Med Sci* 2024, **54**(5):1154-1164.10.55730/1300-0144.5894

53.	Duscharla D, Reddy Kami Reddy K, Dasari C, Bhukya S, Ummanni R: **Interleukin-6 induced overexpression of valosin-containing protein (VCP)/p97 is associated with androgen-independent prostate cancer (AIPC) progression**. *Journal of Cellular Physiology* 2018, **233**(10):7148-7164.<https://doi.org/10.1002/jcp.26639>

54.	Sruthi KK, Ummanni R: **Valosin-Containing Protein (VCP/p97) Mediates Neuroendocrine Differentiation in Prostate Cancer Cells Through Pim1 Signaling Inducing Autophagy**. *The Prostate* 2025, **85**(10):932-946.<https://doi.org/10.1002/pros.24900>
